# DKK3 ameliorates neuropathic pain via inhibiting ASK-1/JNK/p-38-mediated microglia polarization and neuroinflammation

**DOI:** 10.1186/s12974-022-02495-x

**Published:** 2022-06-03

**Authors:** Long-Qing Zhang, Shao-Jie Gao, Jia Sun, Dan-Yang Li, Jia-Yi Wu, Fan-He Song, Dai-Qiang Liu, Ya-Qun Zhou, Wei Mei

**Affiliations:** Department of Anesthesiology and Pain Medicine, Tongji Hospital, Tongji MedicalCollege, Huazhong University of Science and Technology, Wuhan, 430030 Hubei People’s Republic of China

**Keywords:** Neuropathic pain, DKK3, Microglial polarization, Neuroinflammation

## Abstract

**Background:**

Neuropathic pain is a common and severely disabling state that affects millions of people worldwide**.** Microglial activation in the spinal cord plays a critical role in the pathogenesis of neuropathic pain. However, the mechanisms underlying spinal microglial activation during neuropathic pain remain incompletely understood. Here, we investigated the role of Dickkopf (DKK) 3 and its interplay with microglial activation in the spinal cord in neuropathic pain.

**Methods:**

In this study, we investigated the effects of intrathecal injection of recombinant DKK3 (rDKK3) on mechanical allodynia and microglial activation in the spinal cord after spared nerve injury (SNI) in rats by western blot (WB), immunofluorescence (IF), quantitative polymerase chain reaction (qPCR), and enzyme-linked immunosorbent assay (ELISA).

**Results:**

We found that SNI induced a significant decrease in the levels of DKK3, Kremen-1 and Dishevelled-1 (DVL-1) and up-regulated the expression of phosphorylated apoptosis signal-regulating kinase 1 (p-ASK1), phosphorylated c-JUN N-terminal kinase (p-JNK), phosphorylated p38 (p-p38) in the spinal cord. Moreover, our results showed that exogenous intrathecal administration of rDKK3 inhibited expression of p-ASK1, p-JNK, p-p38, promoted the transformation of microglia from M1 type to M2 type, and decreased the production of pro-inflammatory cytokines compared to the rats of SNI + Vehicle. However, these effects were reversed by intrathecal administration of Kremen-1 siRNA or Dishevelled-1 (DVL-1) siRNA.

**Conclusions:**

These results suggest that DKK3 ameliorates neuropathic pain via inhibiting ASK-1/JNK/p-38-mediated microglia polarization and neuroinflammation, at least partly, by the Kremen-1 and DVL-1 pathways.

**Supplementary Information:**

The online version contains supplementary material available at 10.1186/s12974-022-02495-x.

## Introduction

Neuropathic pain is induced by the injury of the peripheral nerve or central nervous system (CNS) [1–4]. Although there are several mechanisms that were identified previously [5, 6], the underlying mechanisms of the development neuropathic pain is still not fully understood. Currently, few effective therapeutic strategies are available for neuropathic pain in clinic. Therefore, it is urgent for us to investigate the underlying mechanisms of neuropathic pain.

Microglia is macrophage-like cells in the CNS that regulate homeostasis in the brain and spinal cord. Increasing evidence from our lab [7, 8] and others [9–14] suggests that microglia plays a critical role in the pathogenesis of neuropathic pain. It has been proved that peripheral nerve injury induces remarkable microgliosis in the spinal cord dorsal horn [15]. Furthermore, microglia is activated within 24 h of nerve injury via polarization [16]. It is known to that M1 phenotype microglia is a pro-inflammatory phenotype which contributes to neuropathic pain. However, the M2 phenotype, as the alternative path of polarization, exerts an anti-inflammatory effect [17]. Moreover, multiple molecules and signaling pathways participate in microglial activation during neuropathic pain, such as MAPK signaling pathway [18, 19]. Specially, phosphorylation of p38 MAPK is increased and highly restricted to spinal microglia after nerve injury [20]. A plenty of studies have demonstrated that pharmacological inhibition of p38 MAPK activity or knockdown of the p38α isoform dampen the development of mechanical allodynia in various models of neuropathic pain [18, 20, 21]. Furthermore, activated microglial cells can release pro-inflammatory cytokines including interleukin 1β (IL-1β), tumor necrosis factor α (TNF-α), and interleukin 6 (IL-6), which contributed to the development of neuropathic pain [4, 10, 22, 23]. Moreover, multiple studies have demonstrated that switching microglia from M1 phenotype to M2 phenotype is a promising therapy in neuropathic pain treatment [17, 24–26].

DKK3, a secretory glycoprotein, plays a vital role in promoting cell survival by suppressing superoxide-producing enzyme and suppress inflammation [27, 28]. It has been proved that DKK3 antagonizes Wnt signaling by interacting with low-density lipoprotein receptor-related protein (LRP) 5/6 in a complex and context-dependent manner [29]. Kremen-1 is a novel transmembrane receptor which function is Wnt inhibitory by removing LRP5/6 from the cell surface via clathrin-mediated endocytosis [30]. DVL-1 is a central component protein that relays Wnt signaling in both canonical and non-canonical pathways whose activity and stability are strictly controlled [31]. A study has demonstrated that DKK3 attenuated microglia activation and neuroinflammation via inhibiting JNK/AP-1/ caspase 1 mediated by Kremen-1 and DVL-1 in mice following intracerebral hemorrhage (ICH) [32]. Moreover, it has been found that DKK3 ameliorates inflammation via inactivation of ASK1/JNK/p38 signaling in myocardial infarction [27]. ASK1, also called mitogen-activated protein kinase kinase kinase 5 (MAP3K5), is a member of MAP kinase kinase kinase family, which activates JNK and p38 in a Raf-independent fashion [33, 34]. Recently, a study reported that inhibition of ASK1/JNK/p38 signaling attenuated neuroinflammation and ameliorated neuropathic pain induced by chronic constrictive injury (CCI) [35].

At present, the mechanisms by which DKK3 modifies the microglial polarization and neuroinflammation during neuropathic pain are unclear. We hypothesized that DKK3 may promote the transformation of microglia from M1 type to M2 type, dampen neuroinflammation in the spinal dorsal horn and attenuate mechanical allodynia in neuropathic pain rats caused by SNI.

## Material and methods

### Animals

Male Sprague–Dawley rats (200–240 g) were supplied by Tongji hospital, Tongji Medical College, Huazhong University of Science and Technology, Wuhan, China. The rats were housed under controlled conditions (temperature: 22–25℃, relative humidity: 45–65%, and 12-h light to dark cycle, with food and water ad libitum). All experiments were approved by the Experimental Animal Care and Use Committee of Tongji hospital, Tongji Medical College, Huazhong University of Science and Technology. All experiments were conducted in accordance with the National Institutes of Health Guidelines for the Care and Use of Laboratory Animals, and the ARRIVE Guidelines for Reporting Animal Research.

### Establishment of neuropathic pain model

A rat model of neuropathic pain was induced by SNI as described previously [36–38]. Briefly, the rats were anesthetized with 2.5% isoflurane, the right sciatic nerve and its three branches (the common peroneal, tibial, sural nerves) were exposed. Then, the common peroneal and tibial nerves were ligated. The distal to the ligation was sectioned, cutting 2 to 4 mm of the distal nerve stump. For the sham-surgery rats, the sciatic nerve was exposed without ligation.

### Mechanical allodynia

To measure mechanical allodynia, paw withdrawal threshold (PWT) in response to von Frey filament stimuli was assessed as described previously [39]. In brief, the rats were placed in individual plastic boxes on a metal mesh floor and allowed to habituate for 30 min. The von Frey filaments (1, 1.4, 2, 4, 6, 8, 10, and 15 g) were applied for up to 6 s per filament to the mid-plantar of the right hind paw. The positive response was defined as the sudden paw withdrawal, licking, and shaking. When a positive response was appeared, the paw was retested after a 5-min rest, starting with the next descending von Frey filament. When no response occurred, the next higher filament was applied. The lowest amount of force required to elicit a positive response was recorded as the PWT (in grams). All of the behavioral tests were performed by an investigator who was blinded to the experimental design.

### Open-field test (OFT)

The OFT was used for locomotor activity measurement, which was performed as previously described [14, 40]. Briefly, motor activity was tested in an open field (60 × 60 × 30 cm). Each rat was placed in the center of the open field and was allowed to move freely for 5 min. A video camera was located 100–120 cm above the floor for behavior recording. Following the completion of a test, 75% alcohol was used to clean the arena. Locomotor behavior was analyzed by counting the total distance and average speed.

### Intrathecal catheterization

As described previously [39], intrathecal (i.t.) catheters was carried out 5 days prior to the establishment of SNI models. Briefly, the rats were anesthetized using isoflurane (2.5%). Then, the PE10 polyethylene catheters (inner diameter 0.3 mm, outer diameter 0.6 mm) were inserted from L5-L6 spinous processes. The correct position of the catheter was verified by a tail flick response immediately after inserting the catheter and further confirmed using an i.t. injection of 2% lidocaine. Animals exhibiting motor dysfunction were excluded from the experiments. No animal was excluded in this study.

### Drug administration

rDKK3 (SRP6268, Sigma-Aldrich, MO, USA) was dissolved in saline. The choice of solvent is based on previous study [32]. During administration with rDkK3, we prepared fresh rDKK3 solution every day and gave it to animals within 15 min. In this study, we aimed to investigate a purely spinal mechanism. Therefore we chose intrathecal injection and the dosage of rDKK3 was determined by our preliminary experiments. To determine whether a single dose of rDKK3 could attenuate established mechanical allodynia in SNI rats, rDKK3 (10, 30, or 50 μg, i.t.) was given on day 7 after surgery. The behavioral test was conducted before rDKK3 injection, and 1, 2, 4, and 6 h after the injection. To determine whether repeated injection of rDKK3 could reverse mechanical allodynia in SNI rats, rDKK3 (30 μg, i.t.) was given once daily for five consecutive days starting from day 7. The behavioral test was performed on day 6 and 2 h after rDKK3 injection each day. To determine whether early treatment with rDKK3 could suppress the development of mechanical allodynia in SNI rats, rDKK3 (30 μg, i.t.) was given once daily for five consecutive days starting from day 1 after the surgery. The behavioral test was conducted before the operation and 2 h after rDKK3 injection on day 3, 7, 8, 9, 10 and 14 after SNI. To identify whether Kremen-1 and DVL-1 are involved in DKK3 regulating microglia polarization and alleviating neuropathic pain in SNI rats, rDKK3 (30 μg, i.t.) was given once daily for five consecutive days starting from day 7 after the operation. The behavioral test was conducted before the operation, and on day 3, 5, 7, 8, 9, 10 and 11 after SNI.

### siRNA transfection

siRNAs were synthesized by Tsingke Biotechnology (Beijing, China). siRNA duplexes that specifically targeted Kremen-1 was: sense 5’-CCUCUCGCAUCCAUUUCAATT-3’, and anti-sense 5’-UUGAAAUGGAUGCGAGAGGTT-3’. siRNA duplexes that specifically targeted DVL-1 was: 5’-CCGAGAUGGAAUGGACAAUTT-3’, and anti-sense 5’-AUUGUCCAUUCCAUCUCGTT-3’. Scramble siRNA was synthesized by a scrambled sequence of nucleotides as a control siRNA, scramble siRNA was: sense 5’-UUCUCCGAACGUGUCACGUTT-3’, anti-sense 5’-ACGUGACACGUUCGGAGAATT-3’. The siRNA was dissolved in RNase-free water at 1 μg/μl and mixed with the transfection reagent branched polyethyleneimine (PEI; Sigma-Aldrich) and 5% glucose for 10 min at room temperature before use. The Kremen-1 siRNA, DVL-1 siRNA or scramble siRNA was administrated via i.t. in 1, 3, 5, 7 days after nerve injury.

### Enzyme-linked immunosorbent assay analysis (ELISA)

Rats were euthanized with CO_2_, the L4–L6 spinal cord segments were quickly excised and homogenized in phosphate-buffered saline (PBS). The supernatant was collected by centrifugation at 15,000*g* at 4 °C for 60 min, and was analyzed using rat IL-1β (Cat#: 88-6010-22; Invitrogen; Carlsbad, CA, USA), TNF-α (Cat#: 88-7340-22; Invitrogen), and IL-6 (Cat#: 88-50625-22; Invitrogen) ELISA kits, according to the manufacturer’s instructions.

### Western blot analysis

Rats were euthanized with CO_2_, the L4–L6 spinal cord segments were quickly excised. Then, the spinal cord was homogenized in an ice-cold mixture of radioimmunoprecipitation assay lysis buffer, phosphatase inhibitor, and phenylmethylsulfonyl fluoride (Boster Biological Technology, Wuhan, Hubei, China), and then centrifuged at 12,000 rpm at 4 ℃ for 30 min. The supernatants were collected, and the protein concentration was determined using a BCA protein assay kit (Cat#: AR0146, Boster Biological Technology). The proteins were boiled at 95 °C in a loading buffer for 10 min. Equivalent amounts of samples (30 μg protein) were separated using 10% sodium dodecyl sulfate–polyacrylamide gels electrophoresis and transferred onto polyvinylidene fluoride membranes (Cat# IPVH00010; Millipore, Billerica, MA, USA). After blocking with 5% bovine serum albumin in Tris-buffered saline and Tween 20 (0.1%) (TBST) for 2 h at room temperature (RT), the membranes were incubated overnight at 4 ℃ with rabbit anti-β-actin antibody (1:5000, Cat# AC026; ABclonal, Woburn, MA, USA); rabbit anti-DKK3 antibody (1:1000, Cat# ab186409; Abcam, Cambridge, UK); rabbit anti Kremen-1 antibody (1:1000, Cat# ab211285; Abcam); rabbit anti DVL-1 antibody (1:1000, Cat# ab233003; Abcam); rabbit anti-ASK1 antibody (1:1000; Cat# A3271; ABclonal); rabbit anti-p-ASK1(1:1000; Cat# 3764; Cell Signaling Technology, Danvers, MA, USA); rabbit anti-JNK antibody (1:1000; Cat# 9258; Cell Signaling Technology); rabbit anti-p-JNK (1:1000; Cat# 4668; Cell Signaling Technology); rabbit anti-p38 antibody (1:1000; Cat# A5049; ABclonal); rabbit anti-p-p38 antibody (1:1000; Cat# 4511; Cell Signaling Technology); goat anti-ionizedcalcium-binding adapter molecule 1(Iba-1, microglial marker) antibody (1:500; Cat# ab5076; Abcam); rabbit anti-CD16 antibody (1:1000; Cat# ab211151; Abcam); rabbit anti-CD86 antibody (1:1000; Cat# ab112490; Abcam); rabbit anti-iNOS antibody (1:1000; Cat#18985-1-AP; Proteintech, Chicago, IL, USA); rabbit anti-Arg1 antibody (1:1000; Cat# A4923; ABclonal); rabbit anti-CD206 antibody (1:500; Cat# ab64693; Abcam); rabbit anti-IL-10 antibody (1:500; Cat# A12255; ABclonal); rabbit anti-IL-1β antibody (1:1000; Cat# AF4006; Affinity Bioscinence; OH; USA); rabbit anti-TNF-α antibody (1:500; Cat# ab205587; Abcam); rabbit anti-IL-6 antibody (1:500; Cat# A0286; ABclonal). The membranes were then washed in TBST and incubated with horseradish peroxidase-conjugated goat anti-rabbit antibody (1:5000; Cat# A21020; Abbkine, Wuhan, Hubei, China), or donkey anti-goat antibody (1:5000; Cat# AS031; ABclonal) for 2 h at RT. The bands were finally visualized with SuperLumia ECL Plus HRP Substrate Kit (Cat# K22030; Abbkine) and then detected using a computerized image analysis system (Bio-Rad, ChemiDoc XRS + , USA). The intensity of protein blot was quantified using System with Image Lab software (Bio-Rad Laboratories), normalized to loading control β-actin antibody and expressed as the fold of control. The blot density of sham, sham + Vehicle, or scramble siRNA groups was set as 1.

### Flow cytometry

As described previously [41], to isolate microglia, spinal cord tissues were removed and minced with scissors in ice‐cold Dulbecco’s modified Eagle medium (DMEM) (Invitrogen). Thereafter, a monocular suspension was formed by digesting with 0.25% trypsin (Invitrogen) in a water bath at 37 °C for 30 min, and then were co-stained for CD86-FITC (an M1 microglia biomarker; 1:200; Cat# 555018; BD Biosciences, San Jose, CA, USA) and CD206-APC (an M2 microglia biomarker; 1:200; Cat# 550889; BD Biosciences) for 45 min at room temperature following the manufacturer’s instructions. The samples were detected using NovoCyte 2040R (ACEA Biosciences) and then analyzed by FlowJo software v.7.6.1.

### Immunofluorescence

Under deep anesthesia with isoflurane (2.5%), the rats were perfused intracardially with 0.1 M PBS, followed by 4% ice-cold paraformaldehyde (PFA) in PBS. Then the L4-L6 spinal cord was removed and post-fixed in 4% PFA for 4 h, and subsequently dehydrated in 30% sucrose solution overnight at 4 ℃. The collected spinal cord samples were sectioned to 20 μm thickness in a cryostat (CM1900, Leica, Wetzlar, Germany). For single immunofluorescent staining, the sections were blocked with 5% donkey serum and 0.3% Triton X-100 for 1 h at RT, and then incubated with rabbit anti-DKK3 antibody (1:50, Cat# ab186409; Abcam); rabbit anti Kremen-1 antibody (1:50, Cat# ab211285; Abcam); rabbit anti DVL-1 antibody (1:50, Cat# ab233003; Abcam); rabbit anti-p-ASK1(1:50; Cat# 3764; Cell Signaling Technology); goat anti-Iba-1 (1:100; Cat# ab5076; Abcam) overnight at 4 ℃. After washing 3 times in PBS, the sections were incubated with CoraLite594-conjugated donkey anti-rabbit secondary antibody (1:100; Cat# SA00013-8; Proteintech) or FITC-conjugated affinipure donkey anti-goat secondary antibody (1:50; Cat# SA00003-3; Proteintech) for 2 h at RT and stained with DAPI for 5 min. For double-immunofluorescence, the sections were blocked with 5% donkey serum and 0.3% Triton X-100 for 1 h at RT, and then incubated with a mixture of rabbit anti-DKK3 antibody (1:50, Cat# ab186409; Abcam), or rabbit anti Kremen-1 antibody (1:50, Cat# ab211285; Abcam), or rabbit anti DVL-1 antibody (1:50, Cat# ab233003; Abcam), or rabbit anti-p-ASK1 antibody (1:50; Cat# 3764; Cell Signaling Technology), or rabbit anti p-JNK antibody (1:50; Cat# ab131499; Abcam), and mouse anti-GFAP antibody (1:100; Cat# 3670; Cell Signaling Technology) or mouse anti-neuronal nuclei antibody (NeuN, neuronal marker) antibody (1:50; Cat# ab104224; Abcam) or goat anti-Iba-1antibody (1:50; Cat# ab5076; Abcam) overnight at 4 ℃. After washing 3 times in PBS, the sections were incubated with a mixture of CoraLite594-conjugated donkey anti-rabbit secondary antibody (1:100; Cat# SA00013-8; Proteintech) and CoraLite488-conjugated donkey anti-mouse secondary antibody (1:50; Cat# SA00013-5; Proteintech) or FITC-conjugated affinipure donkey anti-goat secondary antibody (1:50; Cat# SA00003-3; Proteintech) for 2 h at RT and stained with DAPI for 5 min. Images were captured using a fluorescence microscope (BX51, OLYMPUS, Japan). As described previously [42–45], immunohistochemistry analysis was calculated by using ImageJ software (National Institutes of Health, Bethesda, MD, USA) and averaged per mouse, and each group included 6 animals. The numbers of DKK3, Kremen-1, DVL-1, p-ASK1, Iba-1-positive cells in the spinal dorsal horn were counted in a 500 μm × 500 μm measuring frame. We counted every third sections (40 μm apart) to avoid counting the same cell in more than one section. The cell counts were then used to determine the total number of positive cells per square millimeter. Cells double-labeled for DKK3, or Kremen-1, or DVL-1, or p-ASK1, or p-JNK, and the cell-specific markers NeuN, Iba-1 and GFAP were quantified too in sham and SNI 7d groups. We recorded the number of cells double-labeled with DKK3, or Kremen-1, or DVL-1, or p-ASK1, or p-JNK, and a cell-specific marker in a 500 μm × 500 μm measuring frame in the spinal dorsal horn. The cell counts were then used to determine the total number of double-positive cells per square millimeter.

### Quantitative real-time PCR

In brief, total RNAs were isolated with RNA isolator total RNA extraction reagent (Vazyme, R401-01) according to the manufacturer’s instructions. cDNA was synthesized using PrimeScipt RT Master Mix (Takara, RR036A). Ten microliters of qPCR reactions were prepared from 5 μl Premix Ex TaqII (Takara, RR820A), 0.5 μl primer (final concentration 10 nM), 2 μl DEPC water, and 2 μl cDNA. Reactions were run in a LightCycler 480 qPCR instrument (Roche) using the standard conditions 95 °C for 5 min, 40 cycles (95 °C for 15 s, 56 °C for 30 s, and 72 °C for 30 s) plus melting curve. Relative levels were quantified with the 2-ΔΔCT method that was normalized to the sham group. The primers used were as follows: β-actin forward: 5’-CCCATCTATGAGGGTTACGC-3’, β-actin reverse: 5’-TTTAATGTCACGCACGATTTC-3’; DKK3 forward: 5’-CCCCGACGGCCACTTGGACTC-3’, DKK3 reverse: 5’-GCCGCTTCTTCCGCCTCCATCT-3’; Kremen1 forward: 5’-CGGGCACCAGTAAGACGTCCAACA-3’, Kremen1 reverse: 5’-TGCCTCCCCGTGCTTCCAGTAGTC-3’; DVL-1 forward: 5’-TCGGGGTGGTGAAGGAGGAGATCT-3’, DVL-1 reverse: 5’-CCCCAATGCCGCCTGTCCTCTC-3’.

### Experimental designs and animal groups

**Fig. 1:**
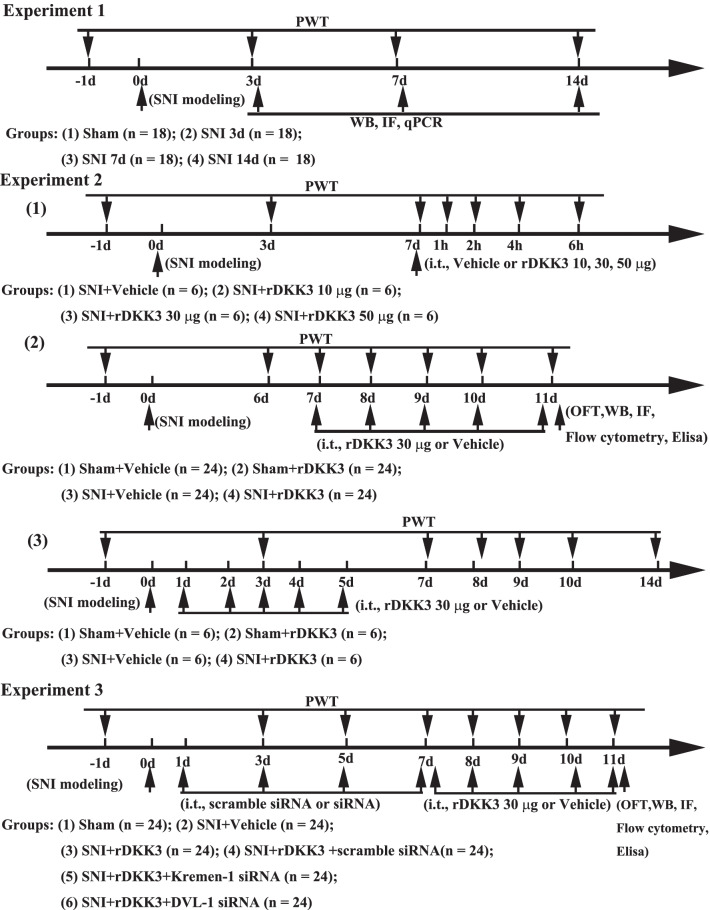
Experimental designs and animal groups. Experiment 1: Changes in mechanical allodynia; the expression of DKK3, Kremen-1, DVL-1, and ASK-1/JNK/p38 MAPK pathway after SNI in rats. Experiment 2: The effects of exogenous rDKK3 administration on mechanical allodynia, microglia polarization and neuroinflammation induced by neuropathic pain. Experiment 3: Kremen-1 siRNA or DVL-1 siRNA abolished the effects of rDKK3 on mechanical allodynia, microglia polarization and neuroinflammation caused by neuropathic pain

In the present study, all rats were randomly assigned to the following three separate experiments which are shown in the timeline of experimental design. The experimental groups and number of animals used in experiments are listed in Fig. 1.

#### Experiment 1: changes in mechanical allodynia and expression of DKK3, Kremen-1, DVL-1, and ASK-1/JNK/p38 MAPK pathway after SNI in rats

Seventy-two rats were randomly allocated to a sham (18 rats) or SNI group (SNI 3d (18rats); SNI 7d (18 rats); SNI 14d (18 rats)). When the last behavioral measurement was finished, we used WB, qPCR, and IF to detect the expression or location of DKK3, Kremen1, DVL-1 and p-ASK-1/p-JNK/p-p38 in the spinal cord.

#### Experiment 2: the effects of exogenous rDKK3 administration on mechanical allodynia, microglia polarization and neuroinflammation induced by neuropathic pain

- (1) To verify whether a single dose of rDKK3 could attenuate mechanical allodynia, 24 rats were randomly divided into four groups (*n* = 6 per group): SNI + Vehicle; SNI + 10 μg rDKK3; SNI + 30 μg rDKK3; SNI + 50 μg rDKK3.
- (2) To determine whether repeated injection of rDKK3 (30 μg) could reverse mechanical allodynia in SNI rats, 96 rats were randomly divided into four groups (*n* = 24 per group): Sham + Vehicle; Sham + rDKK3; SNI + Vehicle; SNI + rDKK3. rDKK3 (30 μg, i.t.) was administrated once daily for five consecutive days starting from day 7 after the surgery. When the last behavioral measurement was finished, the OFT was used to detect the motor function of rats; WB was used to detect the expression p-ASK-1/p-JNK/p-p38; WB and Flow cytometry was used to evaluate the microglia M1/M2 markers; WB and IF was used to detect the expression Iba-1; WB and ELISA was used to examine the pro-inflammatory cytokines.
- (3) To explore whether early treatment with rDKK3 could inhibit the development of mechanical allodynia induced by SNI, rDKK3 (30 μg, i.t.) was given once daily for five consecutive days starting from day 1 after the surgery. Twenty-four rats were randomly divided into four groups (*n* = 6 per group): Sham + Vehicle; Sham + rDKK3; SNI + Vehicle; SNI + rDKK3.

#### Experiment 3: Kremen-1 siRNA or DVL-1 siRNA abolished the effects of rDKK3 on mechanical allodynia, microglia polarization and neuroinflammation caused by neuropathic pain

To identify whether Kremen-1 and DVL-1 are involved in DKK3 regulating microglia polarization and alleviating neuropathic pain in SNI rats, 144 rats randomly divided into six groups (*n* = 24 per group): Sham, SNI + Vehicle, SNI + rDKK3, SNI + rDKK3 + scramble siRNA, SNI + rDKK3 + Kremen-1 siRNA, SNI + rDKK3 + DVL-1 siRNA. The Kremen-1 siRNA, DVL-1 siRNA or scramble siRNA was administrated in 1, 3, 5, 7 days after nerve injury. When the last behavioral measurement was finished, the OFT was used to detect the motor function of rats; WB was used to detect the expression p-ASK-1/p-JNK/p-p38; WB and Flow cytometry was used to evaluate the microglia M1/M2 markers; WB and IF was used to detect the expression Iba-1; WB and ELISA was used to examine the pro-inflammatory cytokines.

### Statistical analyses

Statistical analysis was performed blindly on these independent values. No data points were excluded, and no additional data were subjected to statistical analysis in any experiment. Each animal provided an independent value, and statistical analyses were performed with these numbers. All data are presented as mean ± standard error of the mean (SEM) and analyzed by GraphPad Prism version 6 (Graph Pad Software, San Diego, CA, USA). Two-way repeated measures analysis of variance (ANOVA) followed by Bonferroni’s post hoc test was used for the analysis of the PWT data. One-way ANOVA, followed by Bonferroni’s post hoc test, was used for ELISA, immunofluorescence, qPCR, and western blot data analysis. A *p* < 0.05 was interpreted as statistically significant for all analyses.

## Results

### DKK3, Kremen-1 and DVL-1 are decreased in spinal cord after SNI

**Fig. 2:**
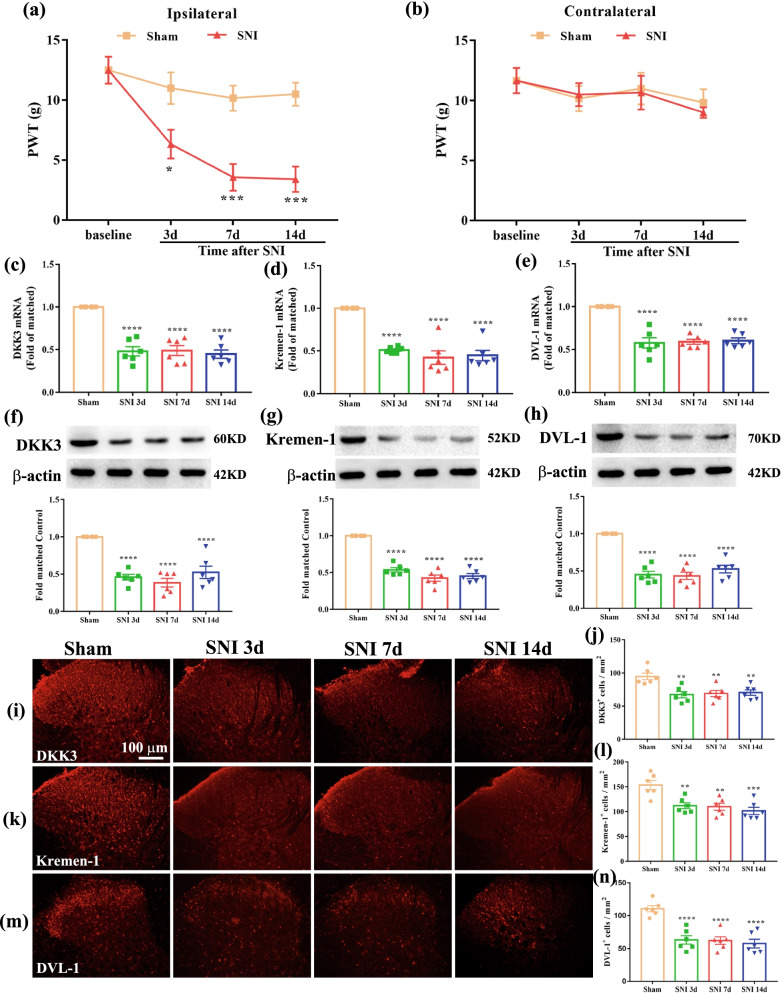
Expression of DKK3, Kremen-1, DVL-1 in the spinal cord following SNI. **a**, **b** Mechanical allodynia evaluated by the paw withdraw threshold (PWT) at baseline and 3, 7 and 14 days after surgery. There is no significant difference regarding the PWT among sham and SNI group at baseline. However, the ipsilateral PWT in SNI rats was markedly decreased from day 3 to day 14 (^*^p < 0.05, ^***^p < 0.001 compared with sham group, *n* = 6 in each group). In contrast, the contralateral PWT in sham and SNI rats had no significant change during the observation period. **c**–**e** qPCR result showed that the mRNA levels of DKK3, Kremen-1, and DVL-1 in SNI rats was markedly decreased from day 3 to day 14 (^****^p < 0.0001 compared with sham group, *n* = 6 in each group). **f**–**h** Western blot results showed that the protein level of DKK3, Kremen-1, and DVL-1 in the spinal cord of rats with SNI was markedly decreased from day 3 to day 14 (^****^p < 0.0001 compared with sham group, *n* = 6 in each group). **i**–**n** Immunofluorescence result indicated that the expression levels of DKK3, Kremen-1, and DVL-1 in SNI rats spinal dorsal horn was markedly decreased from day 3 to day 14 (^**^p < 0.01, ^***^p < 0.001, ^****^p < 0.0001 compared with sham group, *n* = 6 in each group)

As shown in Fig. 2a, there is no significant difference regarding the ipsilateral PWT among sham and SNI rats at baseline. However, the ipsilateral PWT in SNI rats was markedly decreased from day 3 to day 14. In contrast, as shown in Fig. 2b, the contralateral PWT in sham and SNI rats had no significant change during the observation period. These results indicate that SNI successfully induced the development of mechanical allodynia. To explore the potential role of DKK3, Kremen-1 and DVL-1 in neuropathic pain, we examined DKK3, Kremen-1 and DVL-1 expression in spinal cord on day 3, 7, and 14 following SNI. As shown in Fig. 2c–e, DKK3, Kremen-1 and DVL-1mRNA levels were persistently reduced on day 3, 7, and 14 after SNI, when compared with sham control. Western blot revealed that the protein levels of DKK3, Kremen-1 and DVL-1 were significantly decreased on day 3 and sustained on day 14 (Fig. 2f–h). Furthermore, single immunofluorescence further showed SNI-induced lower expression of DKK3, Kremen-1 and DVL-1 in the spinal dorsal horn at 3, 7, and 14 days after SNI, but not in sham control (Fig. 2i–n).

**Fig. 3:**
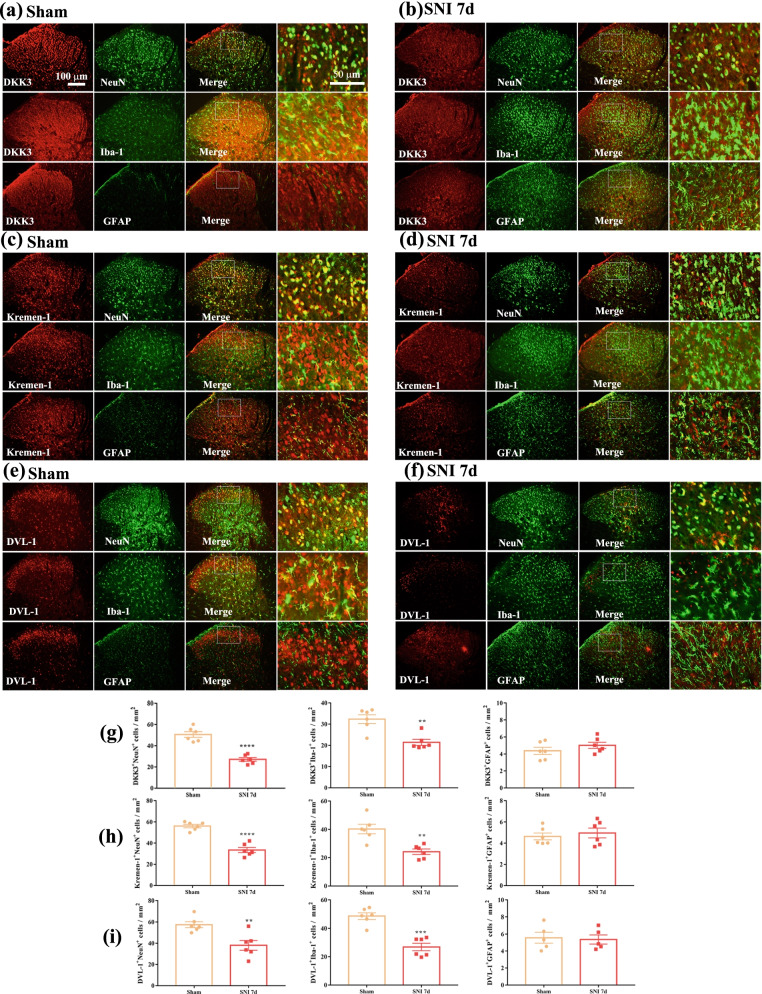
Double-immunofluorescence of DKK3, Kremen-1, DVL-1, and NeuN, Iba1, GFAP in the spinal cord of sham and neuropathic pain rats. **a** Double-immunofluorescence of DKK3 and NeuN, Iba1, GFAP in the spinal cord of sham rats. **b** Double-immunofluorescence of DKK3 and NeuN, Iba1, GFAP in the spinal cord of SNI rats. **c** Double-immunofluorescence of Kremen-1 and NeuN, Iba1, GFAP in the spinal cord of sham rats. **d** Double-immunofluorescence of Kremen-1 and NeuN, Iba1, GFAP in the spinal cord of SNI rats. **e** Double-immunofluorescence of DVL-1 and NeuN, Iba1, GFAP in the spinal cord of sham rats. **f** Double-immunofluorescence of DVL-1 and NeuN, Iba1, GFAP in the spinal cord of SNI rats. **g** Histogram showed that DKK3 co-localization with NeuN, or Iba-1 was decreased in SNI rats (^**^p < 0.01, ^****^p < 0.0001 compared with sham group, *n* = 6 in each group). **h** Histogram showed that Kremen-1 co-localization with NeuN, or Iba-1 was decreased in SNI rats (^**^p < 0.01, ^****^p < 0.0001 compared with sham group, *n* = 6 in each group). **i** Histogram showed that DVL-1 co-localization with NeuN, or Iba-1 was decreased in SNI rats (^**^p < 0.01, ^***^p < 0.001 compared with sham group, *n* = 6 in each group)

To define the type of cells expressing DKK3, Kremen-1 and DVL-1 in the spinal dorsal horn, we performed co-staining of DKK3, Kremen-1 and DVL-1with neuronal marker (NeuN), microglial marker (Iba-1) and astrocytic marker (GFAP). As shown in Fig. 3a–f, in sham-operated rats, DKK3, Kremen-1 and DVL-1 immunoreactivity (IR) was co-stained excessively with NeuN and Iba1, to a lesser extent, with GFAP. We also determined DKK3, Kremen-1 and DVL-1cellular localization at day 7 after SNI, the results demonstrated that the ratio of DKK3, Kremen-1 and DVL-1 co-localization with NeuN or Iba-1 was decreased in SNI 7d group compared with the Sham group (Fig. 3g–i).

### ASK-1/JNK/p38 MAPK signaling pathway is involved in the development of neuropathic pain induced by SNI

**Fig. 4:**
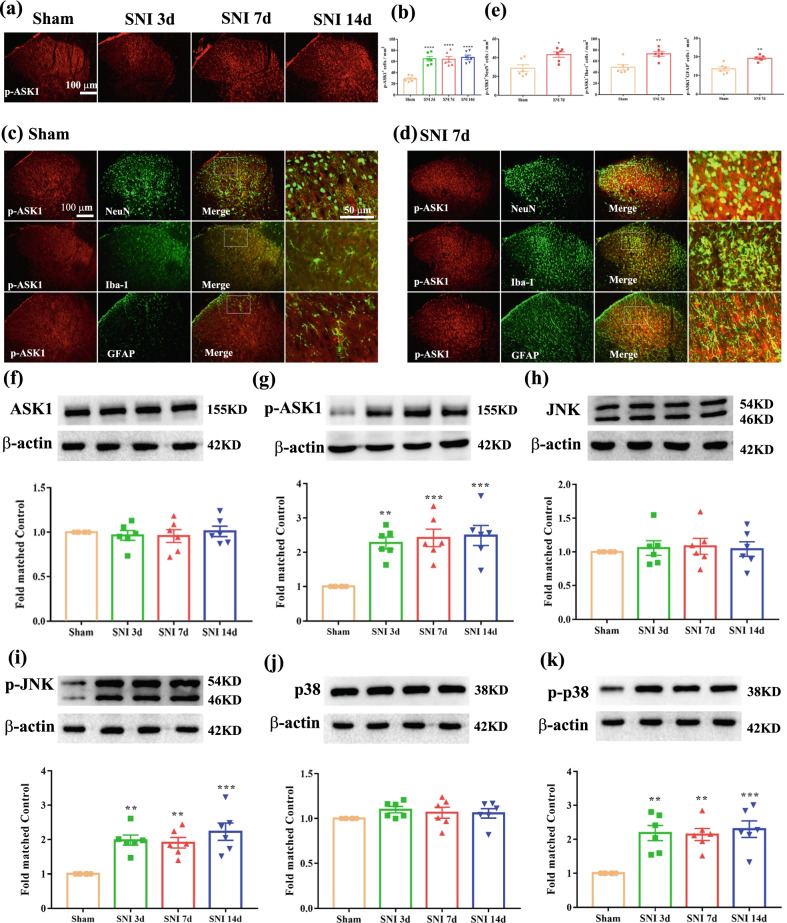
Expression of ASK1/p-ASK1, JNK/p-JNK and p38/p-p38 in the spinal cord following SNI. **a**, **b** Histogram showed the expression level of p-ASK1 in SNI rats spinal dorsal horn was elevated from 3 to 14 days following nerve injury (^****^p < 0.0001 compared with sham group, *n* = 6 in each group). **c** Double-immunofluorescence of p-ASK1 and NeuN, Iba-1, GFAP in the spinal cord of sham rats. **d** Double-immunofluorescence of p-ASK1 and NeuN, Iba-1, GFAP in the spinal cord of SNI rats. **e** Histogram showed that p-ASK1 co-localization with NeuN, or Iba-1, or GFAP was increased in SNI rats (^*^p < 0.05, ^**^p < 0.01 compared with sham group, *n* = 6 in each group). **f**–**g** Western blot result showed that there is no significant difference regarding the protein levels of ASK1 among sham and SNI group, while the protein levels of p-ASK1 in SNI rats was markedly increased from day 3 to day 14 (^**^p < 0.01, ^***^p < 0.001 compared with sham group, *n* = 6 in each group). **h**, **i** Western blot result showed that there is no significant difference regarding the protein levels of JNK among sham and SNI group, while the protein levels of p-JNK in SNI rats was markedly increased from day 3 to day 14 (^**^p < 0.01, ^***^p < 0.001 compared with sham group, *n* = 6 in each group). **j**, **k** Western blot result showed that there is no significant difference regarding the protein levels of p38 among sham and SNI group, while the protein levels of p-p38 in SNI rats was markedly increased from day 3 to day 14 (^**^p < 0.01, ^***^p < 0.001 compared with sham group, *n* = 6 in each group)

Then we examined the protein expression levels of ASK-1, p-ASK-1, JNK, p-JNK, p38, p-p38 in spinal cord. As shown in Fig. 4a, b, single immunofluorescence showed SNI-induced higher expression of p-ASK1 in the spinal dorsal horn at 3, 7, and 14 days after SNI, but not in sham control. To define the type of cells expressing p-ASK1 in the spinal dorsal horn, we performed co-staining of p-ASK1 with neuronal marker (NeuN), microglial marker (Iba-1), and astrocytic marker (GFAP) in sham and SNI 7d group. As shown in Fig. 4c–e, double-immunofluorescence revealed that ratio of p-ASK1 co-localization with Iba-1 was significantly increased in SNI 7d group compared with Sham group. Moreover, it was found that there was no significant difference in ASK-1 (Fig. 4f), JNK (Fig. 4h), p38 (Fig. 4j) protein expression between SNI 3d, 7d, 14d and sham-operated rats. However, the results showed that the protein expression level of p-ASK1 (Fig. 4g), p-JNK (Fig. 4i) and p-p38 (Fig. 4k) were significantly elevated on day 3 and sustained on day 14 after SNI compared with the sham group. Besides, to define the type of cells expressing p-JNK in the spinal dorsal horn, we performed co-staining of p-JNK with NeuN, Iba-1, and GFAP in the sham and SNI 7d group. As shown in Additional file 1: Fig. S1a-e, double-immunofluorescence revealed that ratio of p-JNK co-localization with Iba-1, GFAP was significantly increased in SNI 7d group.

### Analgesic effect of rDKK3 on neuropathic pain rats

**Fig. 5:**
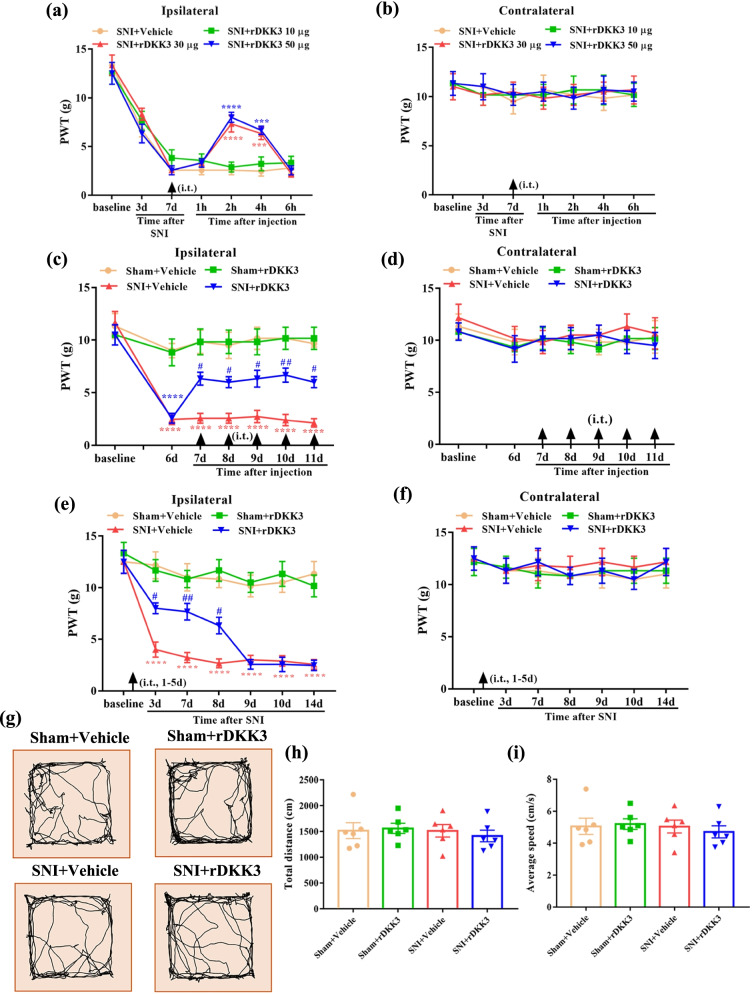
Analgesic effect of rDKK3 on mechanical allodynia in neuropathic pain rats. **a**, **b** A single dose of rDKK3 (30 and 50 μg) markedly increased the ipsilateral PWT in SNI rats, beginning at 2 h and lasted for at least 4 h, and didn’t affect the contralateral PWT (^***^p < 0.001, ^****^p < 0.0001 compared with SNI + Vehicle group, *n* = 6 in each group). **c**, **d** Repetitive injections of rDKK3 (30 μg, i.t.) considerably reversed established mechanical allodynia in the ipsilateral side of SNI rats, and didn’t affect the contralateral PWT (^****^p < 0.0001 compared with Sham + Vehicle group, ^#^*p* < 0.05, ^##^*p* < 0.01 compared with SNI + Vehicle group, *n* = 6 in each group). **e**, **f** The ipsilateral PWT was significantly increased from day 3 to day 8 in rDKK3-treated SNI rats compared with vehicle-treated SNI rats (^****^p < 0.0001 compared with Sham + Vehicle group, ^#^*p* < 0.05, ^##^*p* < 0.01 compared with SNI + Vehicle group, *n* = 6 in each group). And the contralateral PWT in sham and SNI rats treated with Vehicle or rDKK3 had no significant change during the observation period (*p* > 0.05). **g** Horizontal movement traces in the OFT of sham and SNI rats treated with Vehicle or rDKK3. **h**, **i** The OFT result showed that there was no significant difference in the total distance and average speed between the Sham + Vehicle group, Sham + rDKK3 group, SNI + Vehicle group, and SNI + rDKK3 group (*p* > 0.05)

Then, we determined whether rDKK3 could attenuate mechanical allodynia in neuropathic pain rats. To determine whether a single dose of rDKK3 could attenuate established mechanical allodynia in SNI rats, rDKK3 (10, 30, or 50 μg, i.t.) was given on day 7 after surgery. The behavioral test was conducted before rDKK3 injection, and 1, 2, 4, 6 h after the injection. As shown in Fig. 5a, there is no significant change regarding ipsilateral PWT in SNI rats treated with 10 μg ZLN005 compared with SNI + Vehicle group. However, rDKK3 (30 and 50 μg) significantly increased the ipsilateral PWT in SNI rats, peaking at 2 h, and lasted for at least 4 h compared with SNI + Vehicle group. To determine whether repeated injection of rDKK3 could reverse mechanical allodynia in SNI rats, rDKK3 (30 μg, i.t.) was given once daily for five consecutive days starting from day 7. The behavioral test was performed on day 6 and 2 h after rDKK3 injection each day. As shown in Fig. 5c, repetitive injections of rDKK3 (30 μg, i.t.) considerably reversed established mechanical allodynia in SNI rats. To determine whether early treatment with rDKK3 could suppress the development of mechanical allodynia in SNI rats, rDKK3 (30 μg, i.t.) was given once daily for five consecutive days starting from day 1 after the surgery. The behavioral test was conducted before the surgery, and on day 3, 7, 8, 9, 10 and 14 after surgery. As shown in Fig. 5e, the ipsilateral PWT was significantly increased from day 3 to day 8 in rDKK3-treated SNI rats compared with vehicle-treated SNI rats. Moreover, rDKK3 did not affect the PWT on the contralateral side (Fig. 5b, d, f). These results indicate that rDKK3 can markedly attenuate established mechanical allodynia in SNI rats, and also delayed the development of mechanical allodynia in SNI rats. Based on the results from the OFT, there was no significant difference in the total distance and average speed between the sham and SNI rats treated with Vehicle or rDKK3 (Fig. 5g–i). The OFT result indicated that intrathecal injection with rDKK3 did not affect the motor function of rats.

### rDKK3 inhibited the activation of ASK-1/JNK/p38 MAPK signaling pathway in neuropathic pain rats

**Fig. 6:**
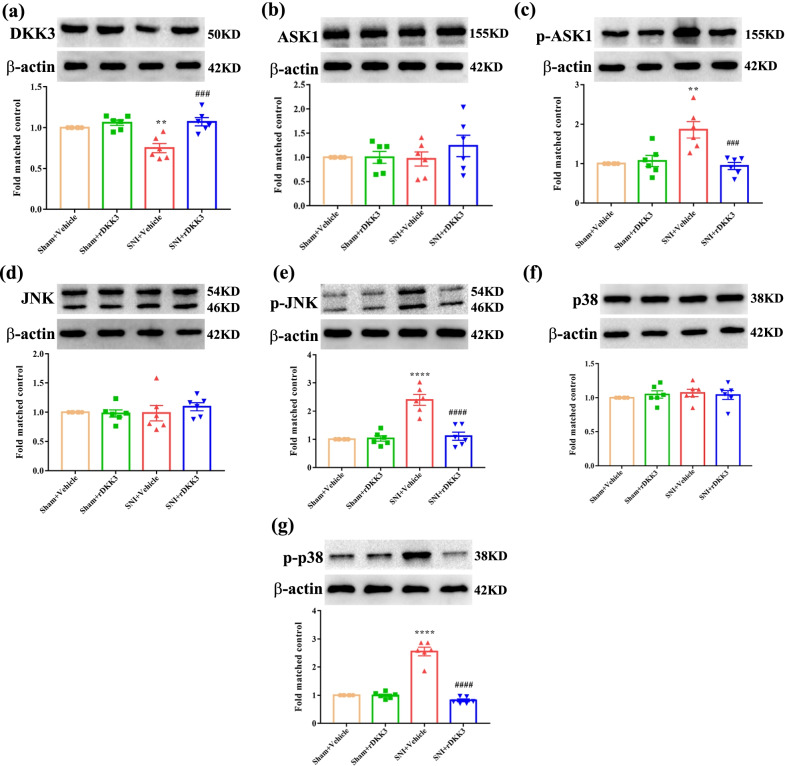
rDKK3 inhibited the activation of ASK1/JNK/p-38 signaling pathway in rats with neuropathic pain. **a** rDKK3 could increase the reduced protein expression level of DKK3 in neuropathic pain rats (^**^p < 0.01 compared with Sham + Vehicle group, ^###^*p* < 0.001 compared with SNI + Vehicle group, *n* = 6 in each group). **b**, **c** Western blot results indicated that the protein level of ASK1 in sham and SNI rats treated with Vehicle or rDKK3 had no significant change, while rDKK3 down-regulated the elevated protein expression level of p-ASK1 induced by neuropathic pain in the spinal cord (^**^p < 0.01 compared with Sham + Vehicle group, ^###^*p* < 0.001 compared with SNI + Vehicle group, *n* = 6 in each group). **d**, **e** Western blot results indicated that the protein level of JNK in sham and SNI rats applied with Vehicle or rDKK3 had no significant change, while rDKK3 down-regulated the increased protein expression level of p-JNK caused by neuropathic pain in the spinal cord (^****^p < 0.0001 compared with Sham + Vehicle group, ^####^*p* < 0.0001 compared with SNI + Vehicle group, *n* = 6 in each group). **f**, **g** Western blot result indicated that the protein level of p38 in sham and SNI rats administrated with Vehicle or rDKK3 had no significant change, while rDKK3 alleviated the increased protein expression level of p-p38 in the spinal cord of rats with neuropathic pain (^****^p < 0.0001 compared with Sham + Vehicle group, ^####^*p* < 0.0001 compared with SNI + Vehicle group, *n* = 6 in each group)

Then, we tested whether treatment with rDKK3 could inhibit the activated ASK-1/JNK/p38 MAPK signaling pathway caused by SNI. Firstly, treatment with rDKK3 attenuated the reduced protein level of DKK3 caused by neuropathic pain (Fig. 6a). Moreover, as shown in Fig. 6b, western blot results displayed that the protein expression level of ASK-1 had no remarkable difference in sham and SNI rats applied with Vehicle or rDKK3. However, rDKK3 could significantly alleviate the increased level of p-ASK-1 in the spinal cord induced by nerve injury compared with the SNI + Vehicle group (Fig. 6c). The results also indicated that there was no significant difference in the protein expression of JNK between sham and SNI rats administrated with Vehicle or rDKK3 (Fig. 6d). But compared with the SNI + Vehicle group, application with rDKK3 dampened the up-regulated levels of p-JNK in the spinal cord of rats with SNI (Fig. 6e). Then, it was found that the level of p38 had no prominent difference in sham and SNI rats treated with Vehicle or rDKK3 (Fig. 6f). Nevertheless, rDKK3 reversed the raised level of p-p38 in the spinal cord of rats with SNI (Fig. 6g).

### rDKK3 promoted the switch of microglia from M1 type to M2 type and suppressed neuroinflammation in neuropathic pain rats

**Fig. 7:**
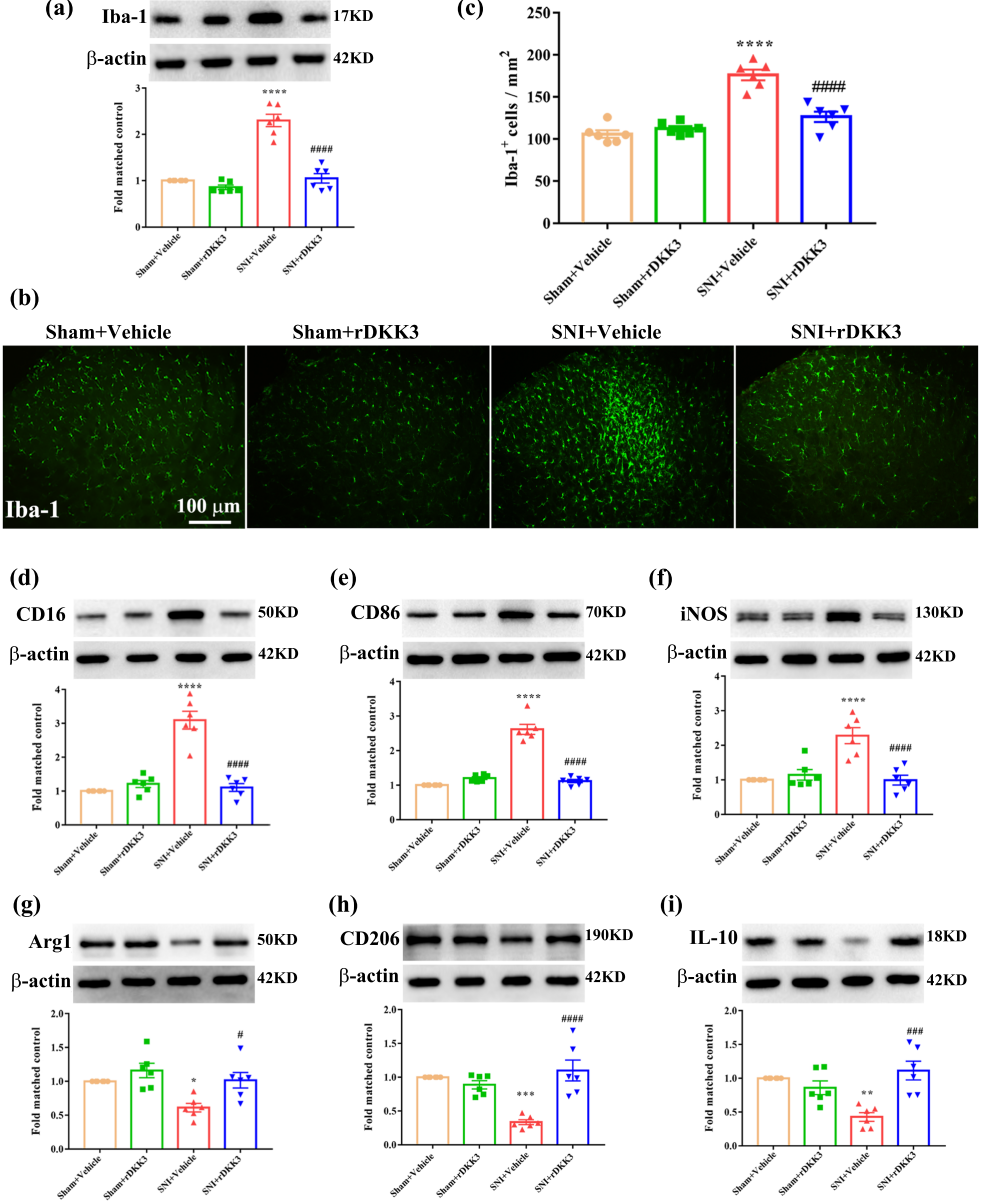
rDKK3 promoted the switch of microglia from M1 type to M2 type in rats with neuropathic pain. **a**–**c** Western blot and immunofluorescence results showed that rDKK3 ameliorated the up-regulated protein expression level of Iba-1 in the spinal cord and suppressed the activated microglia caused by neuropathic pain (^****^p < 0.0001 compared with Sham + Vehicle group, ^####^*p* < 0.0001 compared with SNI + Vehicle group, *n* = 6 in each group). **d**–**f** Western blot results indicated that treatment with rDKK3 attenuated the increased protein expression level of CD16, CD86 and iNOS in the spinal cord induced by neuropathic pain (^****^p < 0.0001 compared with Sham + Vehicle group, ^####^*p* < 0.0001 compared with SNI + Vehicle group, *n* = 6 in each group). **g**–**i** Western blot result indicated that application with rDKK3 up-regulated the decreased protein expression level of Arg1, CD206 and IL-10 in the spinal cord caused by neuropathic pain (^*^p < 0.05, ^**^p < 0.01, ^***^p < 0.001 compared with Sham + Vehicle group, ^#^*p* < 0.05, ^###^*p* < 0.001, ^####^*p* < 0.0001 compared with SNI + Vehicle group, *n* = 6 in each group)

As microglial activation plays an important role in neuropathic pain, we sought to identify whether the increasing spinal DKK3 would alter the status of microglia in spinal dorsal horn after SNI. Microglial activation was demonstrated by the changes of number and morphology in Iba1-positive cells in lamina I–III layers of the dorsal horn. Firstly, we evaluated microglial markers Iba-1 protein expression in the spinal cord, western blot results showed that SNI robustly up-regulated the level of Iba1, and which could be abolished by administration with rDKK3 (Fig. 7a). Moreover, as shown in Fig. 7b, c, SNI triggered radical changes in the morphology and accumulation of Iba1 stained microglia in the spinal dorsal horn. By contrast, SNI induced these alterations of microglia were significantly attenuated by intrathecal injection of rDKK3. Furthermore, we found that rDKK3 prevented SNI-induced M1 microglial polarization and promoted the M2 phenotype. Western blot results showed that application with rDKK3 decreased the protein expression level of M1 markers including CD16 (Fig. 7d), CD86 (Fig. 7e), iNOS (Fig. 7f) and increased the protein expression level of M2 markers including Arg1 (Fig. 7g), CD206 (Fig. 7h), IL-10 (Fig. 7i) in the spinal cord of rats with SNI. Moreover, flow cytometry further confirmed that rDKK3 promoted the switch of microglia from M1 type to M2 type in rats with neuropathic pain (Additional file 2: Fig. S2a-b). Besides, we also observed that rDKK3 could ameliorate neuroinflammation induced by neuropathic pain. Western blot results showed that rDKK3 could reverse the up-regulated levels of pro-inflammatory cytokines including IL-1β (Additional file 3: Fig. S3a), IL-6 (Additional file 3: Fig. S3b), and TNF-α (Additional file 3: Fig. S3c) in the spinal cord. Furthermore, ELISA results further indicated that rDKK3 could alleviate the neuroinflammation induced by neuropathic pain (Additional file 3: Fig. S3d-f).

### The analgesic effect of rDKK3 on neuropathic pain rats was reversed by Kremen-1 siRNA or DVL-1 siRNA

**Fig. 8:**
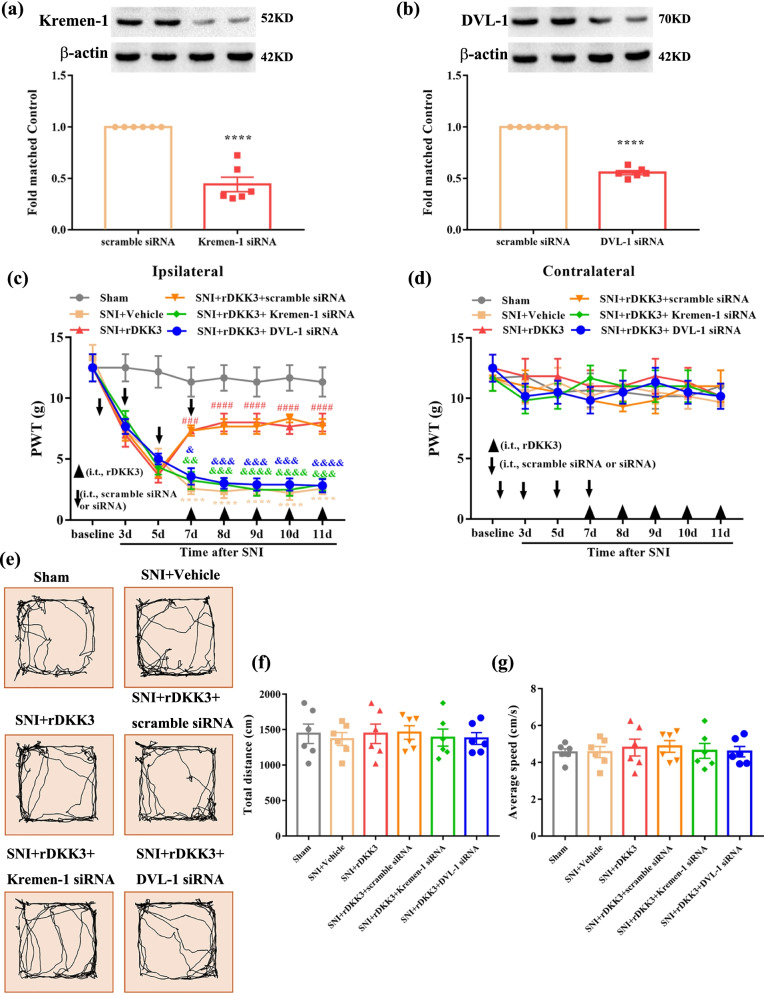
The improved mechanical allodynia was reversed by intrathecal injection with Kremen-1 siRNA or DVL-1 siRNA. **a** Western blot results showed that Kremen-1 siRNA effective down-regulated the protein level of Kremen-1 (^****^p < 0.0001 compared with scramble siRNA group, *n* = 6 in each group). **b** Western blot results showed that DVL-1 siRNA successful down-regulated the protein level of DVL-1 (^****^p < 0.0001 compared with scramble siRNA group, *n* = 6 in each group). **c**, **d** rDKK3 alleviated the decreased ipsilateral PWT induced by neuropathic pain, while application with Kremen-1 siRNA or DVL-1 siRNA both could abolish the analgesic effect of rDKK3 (^****^p < 0.0001 compared with Sham group, ^###^*p* < 0.001, ^####^*p* < 0.001 compared with SNI + Vehicle group, ^&^*p* < 0.05, ^&&^*p* < 0.01, ^&&&^*p* < 0.001 ^&&&&^*p* < 0.0001 compared with SNI + rDKK3 group, *n* = 6 in each group). In contrast, the contralateral PWT in the six groups had no significant change. (e) Horizontal movement traces in the OFT of the Sham group, SNI + Vehicle group, SNI + rDKK3 group, SNI + rDKK3 + scramble siRNA group, SNI + rDKK3 + Kremen-1 siRNA group, and SNI + rDKK3 + DVL-1 siRNA group. **f**, **g** The OFT result showed that there was no significant difference in the total distance and average speed between the Sham group, SNI + Vehicle group, SNI + rDKK3 group, SNI + rDKK3 + scramble siRNA group, SNI + rDKK3 + Kremen-1 siRNA group, and SNI + rDKK3 + DVL-1 siRNA group (*p* > 0.05)

To verify whether Kremen-1 and DVL-1 were participated in the analgesic effect of DKK3, the SNI rats were given rDKK3 in addition to either Kremen-1 siRNA, DVL-1 siRNA, or scramble siRNA. The knockout effect of siRNA was detected by western blot in 11 days following SNI. As shown in Fig. 8a, b, the results showed that Kremen-1 siRNA and DVL-1 siRNA resulted in significant downregulation of Kremen-1 and DVL-1 protein expression. Furthermore, as shown in Fig. 8c, the ipsilateral PWT results indicated that administration with rDKK3 alleviated mechanical allodynia induced by nerve injury compared with SNI + Vehicle group, but this effect was abolished by Kremen-1 siRNA or DVL-1 siRNA. Moreover, Kremen-1 siRNA, DVL-1 siRNA and scramble siRNA all did not affect the PWT on the contralateral side (Fig. 8d). According to the OFT result, there was no significant difference in the total distance and average speed between the Sham group, SNI + Vehicle group, SNI + rDKK3 group, SNI + rDKK3 + scramble siRNA group, SNI + rDKK3 + Kremen-1 siRNA group, and SNI + rDKK3 + DVL-1 siRNA group (Fig. 8e–g). The OFT result demonstrated that intrathecal injection with Kremen-1 siRNA or DVL-1 siRNA did not affect the motor function of rats.

### Both Kremen-1 siRNA and DVL-1 siRNA reversed the effect of rDKK3 on ASK-1/JNK/p38 MAPK signaling pathway in neuropathic pain rats

**Fig. 9:**
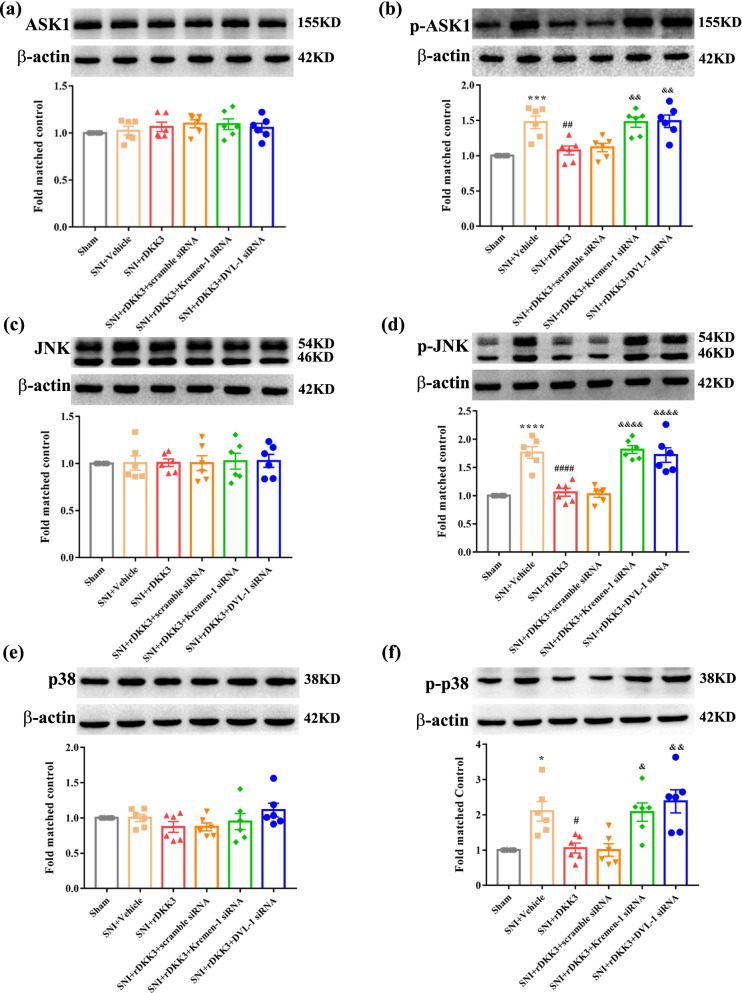
rDKK3 inhibited ASK1/JNK/p38 MAPK signaling pathway via Kremen-1 and DVL-1. **a**, **b** The expression level of ASK1 in the six groups had no significant change. rDKK3 reduced the increased p-ASK1 in neuropathic pain rats, while Kremen-1 siRNA or DVL-1 siRNA both reversed the down-regulated p-ASK1 caused by rDKK3 in neuropathic pain rats (^***^p < 0.001 compared with Sham group, ^##^*p* < 0.01 compared with SNI + Vehicle group, ^&&^*p* < 0.01 compared with SNI + rDKK3 group, *n* = 6 in each group). **c**, **d** The expression level of JNK in the six groups had no significant change. rDKK3 reduced the increased p-JNK in rats with SNI, while Kremen-1 siRNA or DVL-1 siRNA both up-regulated the decreased p-JNK caused by rDKK3 in rats with neuropathic pain (^****^p < 0.0001 compared with Sham group, ^####^*p* < 0.0001 compared with SNI + Vehicle group, ^&&&&^*p* < 0.0001 compared with SNI + rDKK3 group, *n* = 6 in each group). **e**, **f** The expression level of p38 in the six groups had no significant change. rDKK3 dampened the increased p-p38 in neuropathic pain rats, while Kremen-1 siRNA or DVL-1 siRNA both elevated the decreased p-p38 caused by rDKK3 in neuropathic pain rats (^*^p < 0.05 compared with Sham group, ^#^*p* < 0.05 compared with SNI + Vehicle gro up, ^&^*p* < 0.05, ^&&^*p* < 0.01 compared with SNI + rDKK3 group, *n* = 6 in each group)

Then we evaluated the effect of siRNA on ASK-1/JNK/p38 MAPK pathway. As shown in Fig. 9a, western blot results indicated that there was no significantly difference in the protein expression level of ASK1 between the Sham group, SNI + Vehicle group, SNI + rDKK3 group, SNI + rDKK3 + scramble siRNA group, SNI + rDKK3 + Kremen-1 siRNA group, and SNI + rDKK3 + DVL-1 siRNA group. However, application with rDKK3 reduced the increased protein expression level of p-ASK1 in the spinal cord of rats with SNI, while the effect was reversed by intrathecal injection with Kremen-1 siRNA or DVL-1 siRNA (Fig. 9b). Furthermore, the results showed that there was no significantly difference in the protein expression level of JNK between the Sham group, SNI + Vehicle group, SNI + rDKK3 group, SNI + rDKK3 + scramble siRNA group, SNI + rDKK3 + Kremen-1 siRNA group, and SNI + rDKK3 + DVL-1 siRNA group (Fig. 9c). However, treatment with rDKK3 ameliorated the increased level of p-JNK in the spinal cord of rats with SNI, while Kremen-1 siRNA or DVL-1 siRNA dampened the effect (Fig. 9d). Moreover, western blot results implied that there was no significantly difference in the expression level of p38 between the Sham group, SNI + Vehicle group, SNI + rDKK3 group, SNI + rDKK3 + scramble siRNA group, SNI + rDKK3 + Kremen-1 siRNA group, and SNI + rDKK3 + DVL-1 siRNA group (Fig. 9e). But, administration with rDKK3 down-regulated the raised level of p-p38 in the spinal cord of rats with SNI, while Kremen-1 siRNA or DVL-1 siRNA reversed the effect (Fig. 9f).

### Both Kremen-1 siRNA and DVL-1 siRNA reversed the effect of rDKK3 on microglia polarization and neuroinflammation in neuropathic pain rats

**Fig. 10:**
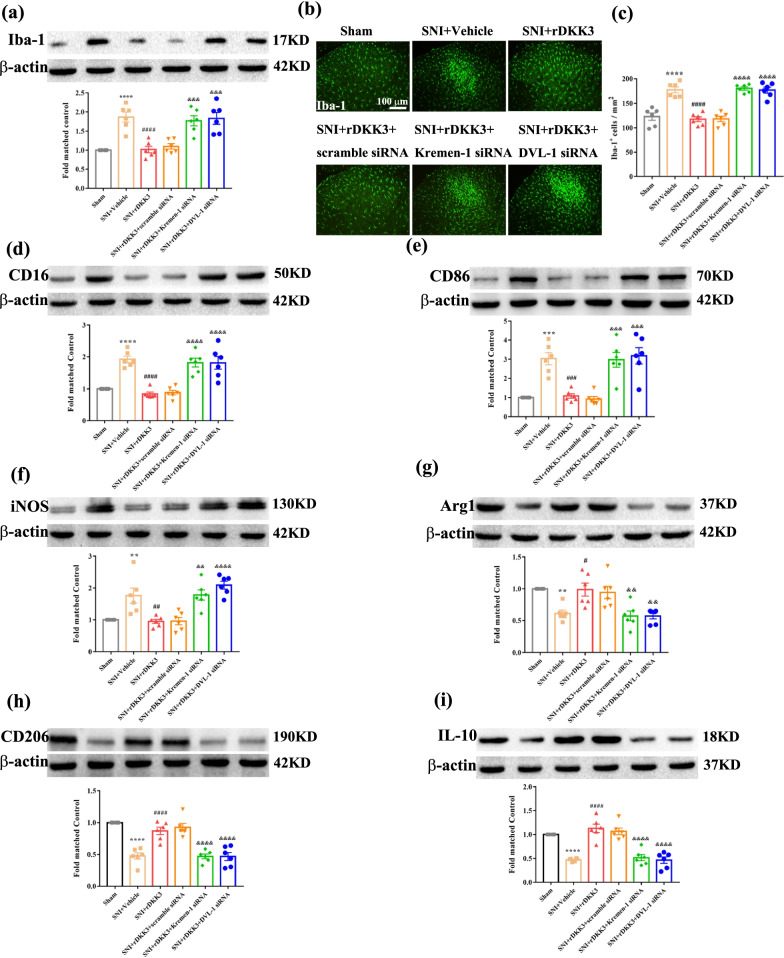
rDKK3 promoted the switch of microglia from M1 type to M2 type in neuropathic pain rats via Kremen-1 and DVL-1. **a**–**c** Western blot and immunofluorescence results showed that rDKK3 ameliorated the up-regulated protein expression level of Iba-1 in the spinal cord and suppressed the activated microglia caused by neuropathic pain, while treatment with Kremen-1 siRNA or DVL-1 siRNA abolished the effect of rDKK3 on microglia activation (^****^p < 0.0001 compared with Sham group, ^####^*p* < 0.0001 compared with SNI + Vehicle group, ^&&&&^*p* < 0.0001 compared with SNI + rDKK3 group, *n* = 6 in each group). **d**–**f** Western blot results indicated that treatment with rDKK3 attenuated the increased protein expression level of CD16, CD86 and iNOS in the spinal cord induced by neuropathic pain, while Kremen-1 siRNA or DVL-1 siRNA abolished the effect of rDKK3 on M1 type biomarkers (^**^p < 0.01, ^***^p < 0.001, ^****^p < 0.0001 compared with Sham group, ^##^*p* < 0.01, ^###^*p* < 0.001, ^####^*p* < 0.0001 compared with SNI + Vehicle group, ^&&^*p* < 0.01, ^&&&^*p* < 0.001, ^&&&&^*p* < 0.0001 compared with SNI + rDKK3 group, *n* = 6 in each group). **g**–**i** Western blot results indicated that application with rDKK3 up-regulated the decreased protein expression level of Arg1, CD206 and IL-10 in the spinal cord caused by neuropathic pain, while administration with Kremen-1 siRNA or DVL-1 siRNA attenuated the effect of rDKK3 on M2 type biomarkers ^(**^p < 0.01, ^****^p < 0.0001 compared with Sham group, ^#^*p* < 0.05, ^####^*p* < 0.0001 compared with SNI + Vehicle group, ^&&^*p* < 0.01, ^&&&&^*p* < 0.0001 compared with SNI + rDKK3 group, *n* = 6 in each group)

Firstly, we evaluated microglial markers Iba-1 protein expression in the spinal cord, western blot results showed that Kremen-1 siRNA and DVL-1 siRNA both could reverse the reduced protein level of Iba-1 caused by rDKK3 (Fig. 10a). Moreover, as shown in Fig. 10b-c, SNI triggered radical changes in the morphology and accumulation of Iba-1 stained microglia in the spinal dorsal horn. SNI induced these alterations of microglia were significantly abolished by intrathecal injection of rDKK3, while administration with Kremen-1 siRNA and DVL-1 siRNA both could abolished the effect of rDKK3 on microglia activation. Moreover, western blot results showed that rDKK3 down-regulated the increased levels of CD16 (Fig. 10d), CD86 (Fig. 10e), iNOS (Fig. 10f) and up-regulated the decreased levels of Arg1 (Fig. 10g), CD206 (Fig. 10h), IL-10 (Fig. 10i) compared with the SNI + Vehicle group, while both knock out Kremen-1 with Kremen-1 siRNA and knock out DVL-1 with DVL-1 siRNA could reverse the effect of rDKK3 on microglia polarization. Flow cytometry further identified that rDKK3 promoted the switch of microglia from M1 type to M2 type in rats with neuropathic pain, while Kremen-1 siRNA and DVL-1 siRNA could abrogate the effect of rDKK3 on microglia polarization (Additional file 4: Fig. S4a-b). In addition to this, western blot (Additional file 5: Fig. S5a-c) and ELISA (Additional file 5: Fig. S5d-f) results showed that Kremen-1 siRNA and DVL-1 siRNA both could reverse the attenuated neuroinflammation in the spinal cord induced by rDKK3 compared with the SNI + rDKK3 group.

## Discussion

In this study, we showed that (1) intrathecal administration of rDKK3 ameliorated established mechanical allodynia, and delayed the onset of mechanical allodynia in SNI rats; (2) intrathecal administration of rDKK3 promoted microglia transformation from M1 type to M2 type, and improved neuroinflammation via inhibiting ASK-1/JNK/p38 MAPK pathway in SNI rats; (3) application with Kremen-1 siRNA or DVL-1 siRNA abolished the effects of rDKK3 on mechanical allodynia, microglia polarization and neuroinflammation in SNI rats. Taken together, our results demonstrated that DKK3 interacted with Kremen1 and DVL-1, which improved mechanical allodynia by promoting microglia transformed from M1 type to M2 type, and ameliorating neuroinflammation via inhibiting ASK-1/JNK/p38 MAPK.

Previous studies have indicated that DKK3 was expressed in in the liver [46], heart [47], kidney [48], and brain [49]. However, our PCR and WB results proved that DKK3 was also expressed in the rat spinal cord. The biological action of DKK3 is complex and still not fully understood. It was reported that DKK3 protects neurons against a variety of toxic insults via mediating vascular endothelial growth factor (VEGF), and DKK3 overexpression substantially alleviated cardiac hypertrophy and fibrosis [27]. Moreover, a study proved that rDKK3 protected mice against ICH via inhibiting ICH-induced inflammation [32]. In our study, PCR, western blot and immunofluorescence results demonstrated that the expression level of DKK3 was decreased in SNI day 3 until day 14 in spinal cord compared with the Sham group. Thereby, in present study, three doses of rDKK3 (10 μg, 30 μg, and 50 μg) were evaluated in the SNI rat model, we showed that intrathecal administration with rDKK3 (30 μg, or 50 μg) had an analgesic effect in rats with neuropathic pain in a dosage independent manner. Furthermore, we also found that early treatment with rDKK3 could delay the onset of mechanical allodynia induced by SNI. Our study found that intrathecal administration with rDKK3 in 1–5 days after SNI could produce analgesic effect up to 8th day. The long analgesic time maintained by continuous administration with rDKK3 is the potential advantage of rDKK3 in the treatment of neuropathic pain. However, the specific mechanism needs to be identified by further study.

Although the specific receptors and related signaling pathways that DKK3 interacts with are still controversial, it has been confirmed that DKK3 interaction with Kremen-1 may affect Wnt signaling [50] and DKK3 immunoprecipitated with Kremen-1, but not lipoprotein receptor-related protein 6 (LRP6), in cancer-associated fibroblasts [51]. In addition to this, a study suggested DVL-1 plays a significant role in the downstream signaling involved in the DKK3 mechanism in endothelial cells [52]. Furthermore, it has been demonstrated that silencing Kremen-1 or DVL-1 with siRNA both reversed the anti-inflammatory effects of DKK3 in ICH mice [32]. DVL-1 is composed of three conserved domains (N-terminal DIX domain, PDZ domain, and a C-terminal DEP domain). The PDZ domain remarkably contributes to protein interactions. This domain binds to the membrane-bound receptor and to other signal transduction molecules in the cytoplasm to distinguish between suitable binding partners [53]. Furthermore, it has been proposed that when DKK3 interacts with the Kremen-1, then the transmembrane receptor Kremen-1 may compete with the canonical signaling pathway for the interaction with DVL-1 and downstream molecules through its PDZ domain [32]. Moreover, this action of DVL-1 is independent on Wnt or its downstream effector β-catenin. Our results also indicated that the improved mechanical allodynia induced by rDKK3 was abolished by Kremen-1 or DVL-1 silencing, which demonstrated that DKK3 attenuate neuropathic pain mediated by Kremen-1 and DVL-1. DKK3 may form a ternary complex with Kremen-1 receptors and DVL-1, thus allowing fine-tuning of downstream signaling.

A plenty of evidence has demonstrated that the activation of microglia is the early event that mediates neuroinflammation, which plays a crucial role in the development of neuropathic pain [54–56]. Therefore, it is necessary to further understand microglial activation for expanding our knowledge regarding the pathogenesis of neuropathic pain. Our results indicated that DKK3, Kremen-1, and DVL-1 were mainly expressed in neurons and microglia in spinal cord dorsal horn, which is consistent with previous studies [32]. Neuropathic pain could decrease the expression of DKK3, Kremen-1, and DVL-1 in microglia, this possibly showed that DKK3 is produced by microglia and stimulates the same microglia mediated by Kremen-1 and DVL-1 to reduce its own activation and acts as a protective molecule. In addition to this, it has been proved that DKK3 could alleviate chronic inflammation in liver [46] and down-regulate the multiple pro-inflammatory cytokines derived from activated microglia in brain [32]. Our study also found that DKK3 could dampen the neuroinflammation caused by neuropathic pain mediated by Kremen-1 and DVL-1.

ASK1/JNK/p38 MPAK signaling pathway was involved in the development of multiple diseases [27, 35, 57]. It has been identified that inactivation ASK1/JNK/p38 MAPK pathway could alleviate neuroinflammation and neuropathic pain induced by chronic constrictive injury (CCI) via inhibiting the activation of microglia [35]. Furthermore, another study demonstrated that DKK3 protected against the development of myocardial infarction (MI)-induced cardiac remodeling via inhibiting the ASK1/JNK/p38 MPAK signaling pathway [27]. Recently, growing evidences have found that ASK1 is expressed in glial cells and contributes to the induction and maintenance of neuroinflammation [35, 57, 58]. It has also been found that inhibition of ASK1 could significantly ameliorate the phosphorylation of JNK and p38, and reduce pro-inflammatory factors released by activated glial cells, which are crucial mediators in the process of pain sensitization [35]. Furthermore, JNK/p38 MAPK pathway played a remarkable role in promoting the polarization of microglia to M1 type after nerve injury. According to previous studies [59, 60], p-JNK is primarily observed in spinal cord astrocytes of nerve injury rats. However, the detection time is 14 days or 21 days after nerve injury in these studies. Our study focused on the early stages of nerve injury. Moreover, previous studies has identified that p-JNK immunoreactivity was increased in spinal microglia of rats with spinal cord injury [61, 62]. Furthermore, our immunofluorescence result showed that most of the increased p-JNK was expressed in astrocytes and a few in microglia at 7 days after nerve injury. Our study firstly demonstrated that DKK3 could promote microglia polarization from M1 type to M2 type via negative regulation of the ASK1/JNK/p38 MAPK signaling pathway in SNI rats, however, these effects of DKK3 were reversed by Kremen-1 siRNA or DVL-1 siRNA.

Our results strongly suggested that the suppression the ASK1/JNK/p38 pathway largely account for the analgesia effects of DKK3. However, one of the major limitations of our study is that we paid special attention to the involvement of DKK3 to Kremen-1 receptor, DVL-1, and ASK-1/JNK/p38 signaling pathway, but other potential mechanisms have not been investigated. DKK3 has been found to inhibit inflammation via the non-canonical Wnt signaling pathway in a model of atherosclerosis [52]. In addition, it has been proved that DKK3 protected against ICH via inhibiting the activation microglia and negative regulating JNK/AP-1 signaling cascades [32]. Another limitation of our study is that there is no evidence of clinical application of DKK3 in treatment of neuropathic pain. It is necessary to accelerate the transformation from basic research to clinical research in future studies. Furthermore, intrathecal injection is a simple and relatively noninvasive means to deliver agents to DRG sensory neurons. So, intrathecal injection with rDKK3 may attenuate mechanical allodynia via interaction with DRG sensory neurons. But, the specific mechanism needs to be further studied.

## Conclusion

**Fig. 11:**
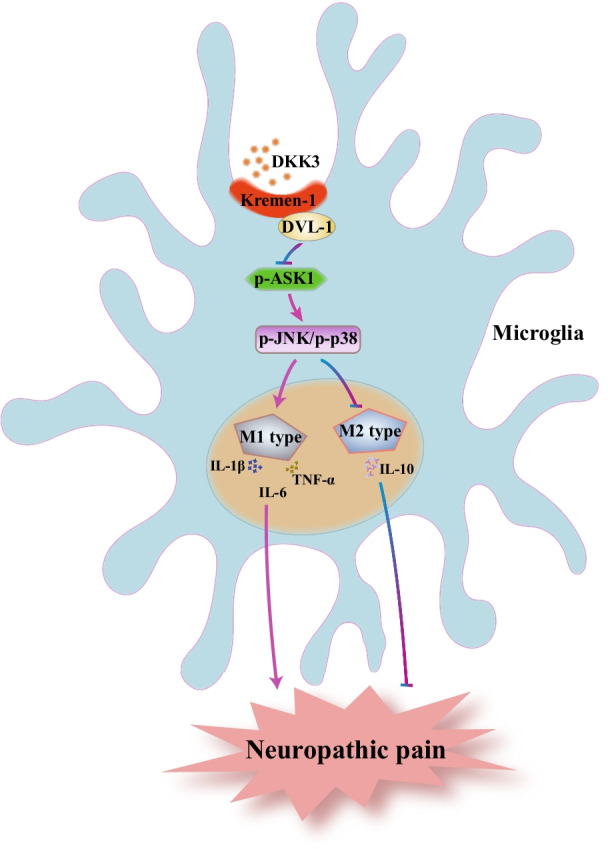
Schematic illustration of the role DKK3 in neuropathic pain

In summary, we demonstrated that DKK3 could attenuate neuropathic pain through suppressing ASK-1/JNK/p38 MAPK signaling pathway, promoting microglia polarization from M1 type to M2 type and improving neuroinflammation mediated by Kremen-1 and DVL-1 in the spinal cord (Fig. 11). Our finding provided a new perspective on microglia polarization involved of DKK3.

## Supplementary Information

**Additional file 1: Fig. S1.** Double immunofluorescence of p-JNK and NeuN, Iba1, GFAP in the spinal cord of sham and neuropathic pain rats. (a) Double immunofluorescence of p-JNK and NeuN, Iba1, GFAP in the spinal cord of sham rats. (b) Double immunofluorescence of p-JNK and NeuN, Iba1, GFAP in the spinal cord of SNI rats. (c-e) Histogram showed that p-JNK co-localization with Iba-1, or GFAP was increased in the SNI 7d group (^*^p < 0.05, ^***^p < 0.001 compared with Sham group, *n* = 6 in each group).

**Additional file 2: Fig. S2.** The Flow cytometry result of the Sham + Vehicle group, Sham + rDKK3 group, SNI + Vehicle group, and SNI + rDKK3 group. (a) Representative dot spot of flow cytometry for microglia. (b) Histogram showed that rDKK3 promoted the switch of microglia from M1 type to M2 type in rats with neuropathic pain (^**^p < 0.01, compared with Sham + Vehicle group, ^##^*p* < 0.01, compared with SNI + Vehicle group, *n* = 6 in each group).

**Additional file 3: Fig. S3.** rDKK3 alleviated neuroinflammation in the spinal cord induced by neuropathic pain. (a-c) Western blot results indicated that administration with rDKK3 reduced the pro-inflammatory cytokines level of IL-1β, IL-6 and TNF-α in the spinal cord caused by neuropathic pain (^*^p < 0.05, ^**^p < 0.01, ^***^p < 0.001 compared with Sham + Vehicle group, ^#^*p* < 0.05, ^###^*p* < 0.001, ^###^*p* < 0.0001 compared with SNI + Vehicle group, *n* = 6 in each group). (d-f) ELISA results showed that rDKK3 suppressed neuroinflammation in the spinal cord caused by neuropathic pain (^***^p < 0.001, ^****^p < 0.0001 compared with Sham + Vehicle group, ^##^*p* < 0.01, compared with SNI + Vehicle group, *n* = 6 in each group).

**Additional file 4: Fig. S4.** The Flow cytometry result of the Sham group, SNI + Vehicle group, SNI + rDKK3 group, SNI + rDKK3 + scramble siRNA group, SNI + rDKK3 + Kremen-1 siRNA group, and SNI + rDKK3 + DVL-1 siRNA group. (a) Representative dot spot of flow cytometry for microglia. (b) Histogram showed that rDKK3 promoted the switch of microglia from M1 type to M2 type in the spinal cord of rats with neuropathic pain, while Kremen-1 siRNA and DVL-1 siRNA could abrogate the effect of rDKK3 on microglia polarization (^**^p < 0.01 compared with Sham group, ^##^*p* < 0.01 compared with SNI + Vehicle group, ^&&&^*p* < 0.001 compared with SNI + rDKK3 group, *n* = 6 in each group).

**Additional file 5: Fig. S5.** The improved neuroinflammation caused by rDKK3 was abolished by Kremen-1 siRNA or DVL-1 siRNA in neuropathic pain rats. (a-c) Western blot results indicated that rDKK3 reduced the pro-inflammatory cytokines level of IL-1β, IL-6 and TNF-α in the spinal cord caused by neuropathic pain, while administration with Kremen-1 siRNA or DVL-1 siRNA up-regulated the decreased pro-inflammatory cytokines level of IL-1β, IL-6 and TNF-α in the spinal cord caused by neuropathic pain (^*^p < 0.05, ^**^p < 0.01 compared with Sham group, ^#^*p* < 0.05, ^###^*p* < 0.001 compared with SNI + Vehicle group, ^&^*p* < 0.05, ^&&^*p* < 0.01, ^&&&^*p* < 0.001, ^&&&&^*p* < 0.0001 compared with SNI + rDKK3 group, *n* = 6 in each group). (d-f) ELISA results showed that the improved neuroinflammation in the spinal cord caused by rDKK3 was abolished by Kremen-1 siRNA or DVL-1 siRNA in rats with neuropathic pain (^***^p < 0.001, ^****^p < 0.0001 compared with Sham group, ^##^*p* < 0.01, ^###^*p* < 0.001 compared with SNI + Vehicle group, ^&&^*p* < 0.01, ^&&&^*p* < 0.001 compared with SNI + rDKK3 group, *n* = 6 in each group).

## Data Availability

The data and materials supporting the conclusions of this study are available from the corresponding author on reasonable request.

## Abbreviations

DKK 3: Dickkopf 3
DVL-1: Dishevelled-1
SNI: Spared nerve injury
WB: Western blot
IF: Immunofluorescence
qPCR: Quantitative polymerase chain reaction
ELISA: Enzyme-linked immunosorbent assay
rDKK3: Recombinant DKK3
p-ASK1: Phosphorylated apoptosis signal-regulating kinase 1
p-JNK: Phosphorylated c-JUN N-terminal kinase
p-p38: Phosphorylated p38
IL-1β: Interleukin 1β
TNF-α: Tumor necrosis factor α
IL-6: Interleukin 6
IL-10: Interleukin 10
MAP3K5: Mitogen-activated protein kinase kinase kinase 5
PWT: Paw withdrawal threshold
