# Supplementary figures and images for: DKK3 ameliorates neuropathic pain via inhibiting ASK-1/JNK/p-38-mediated microglia polarization and neuroinflammation
Source: J Neuroinflammation. 2022 Jun 3;19:129. doi: 10.1186/s12974-022-02495-x (PMC9164405; doi:10.1186/s12974-022-02495-x)

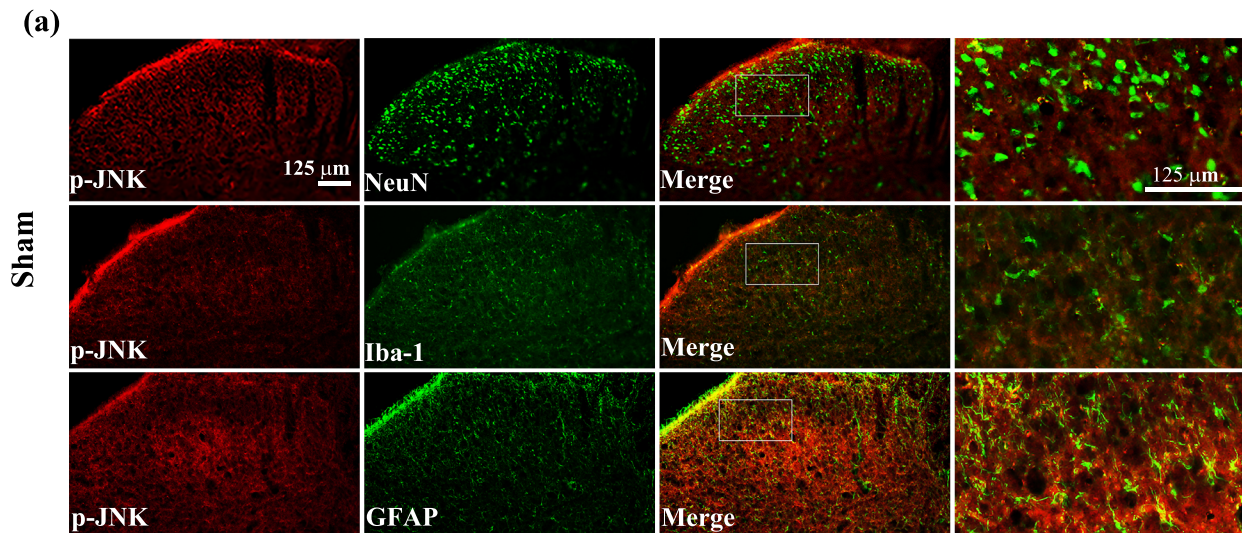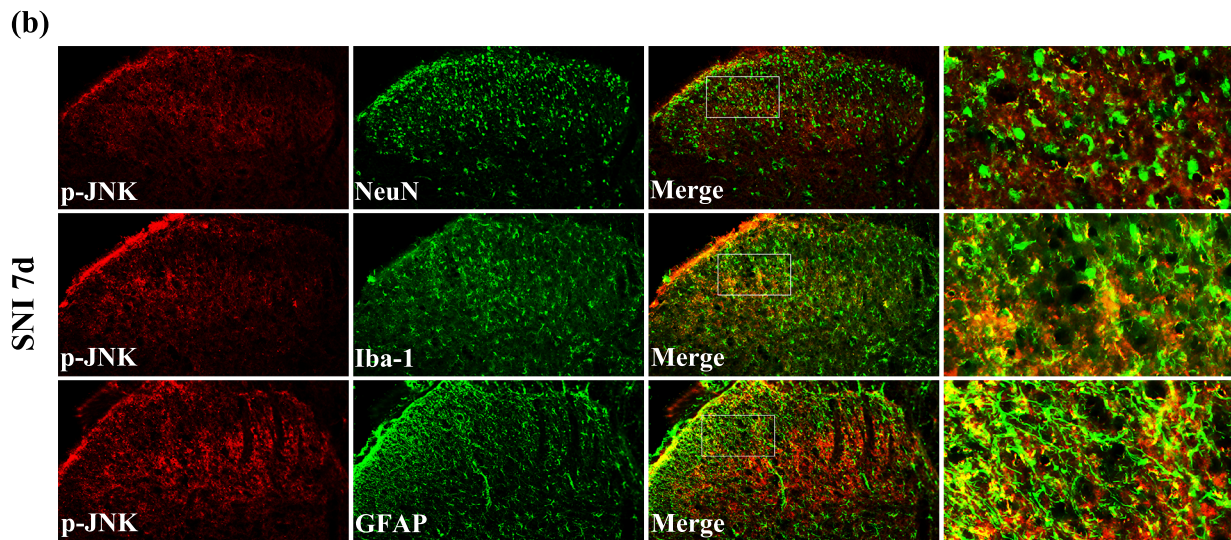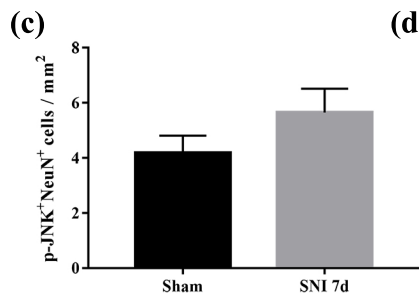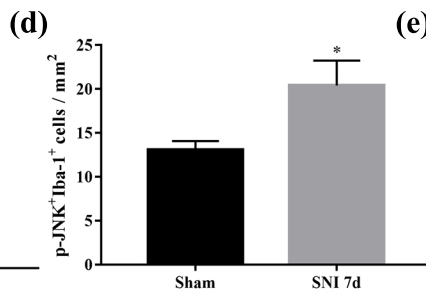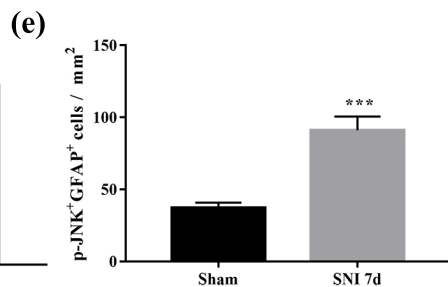

Supplement: Supplementary file 1 — Additional file 1: Fig. S1. Double immunofluorescence of p-JNK and NeuN, Iba1, GFAP in the spinal cord of sham and neuropathic pain rats. (a) Double immunofluorescence of p-JNK and NeuN, Iba1, GFAP in the spinal cord of sham rats. (b) Double immunofluorescence of p-JNK and NeuN, Iba1, GFAP in the spinal cord of SNI rats. (c-e) Histogram showed that p-JNK co-localization with Iba-1, or GFAP was increased in the SNI 7d group (*p < 0.05, ***p < 0.001 compared with Sham group, n = 6 in each group). [file 12974_2022_2495_MOESM1_ESM.pdf]

(a)

CD86

Sham+Vehicle

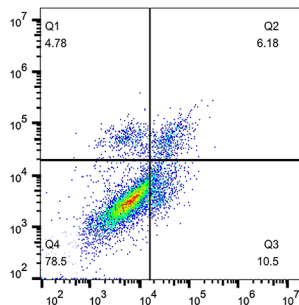

Sham+rDKK3

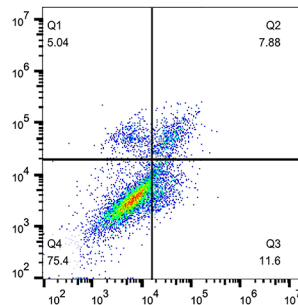

SNI+Vehicle

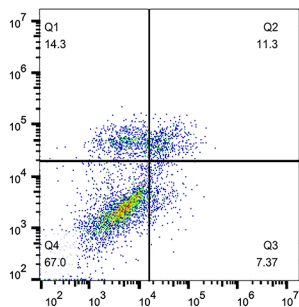

SNI+DKK3

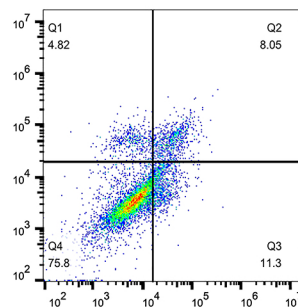

CD206

(b)

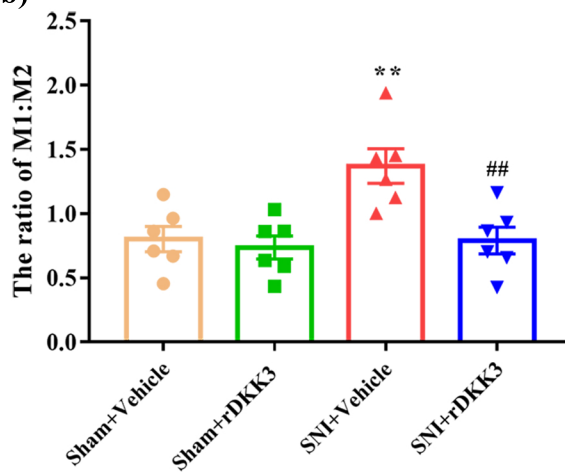

Supplement: Supplementary file 2 — Additional file 2: Fig. S2. The Flow cytometry result of the Sham + Vehicle group, Sham + rDKK3 group, SNI + Vehicle group, and SNI + rDKK3 group. (a) Representative dot spot of flow cytometry for microglia. (b) Histogram showed that rDKK3 promoted the switch of microglia from M1 type to M2 type in rats with neuropathic pain (**p < 0.01, compared with Sham + Vehicle group, ##p < 0.01, compared with SNI + Vehicle group, n = 6 in each group). [file 12974_2022_2495_MOESM2_ESM.pdf]

**(a)**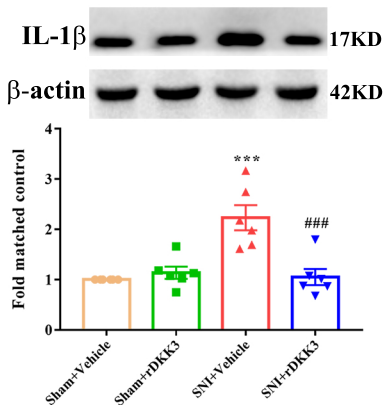**(b)**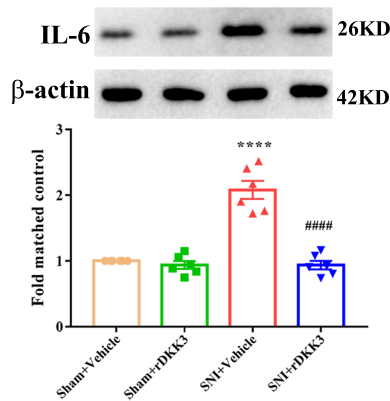**(c)**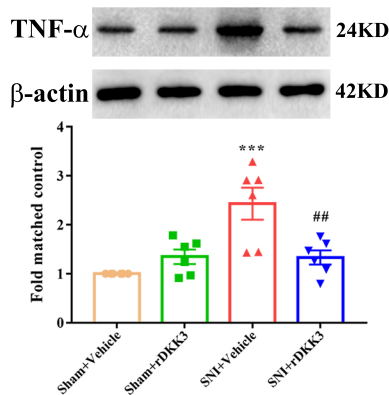**(d)**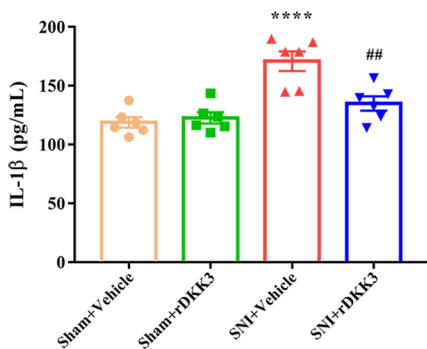**(e)**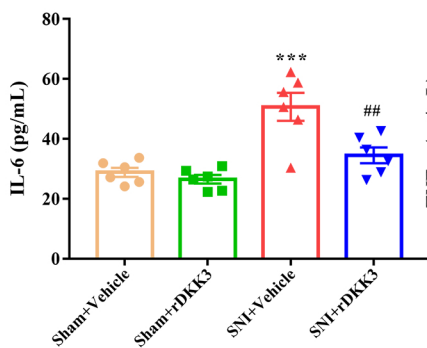**(f)**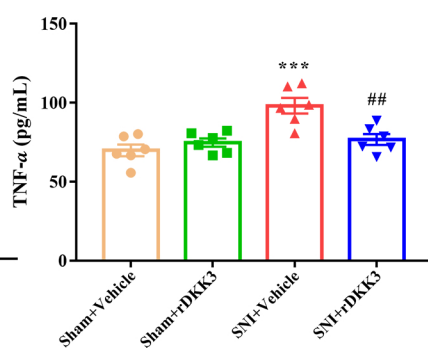

Supplement: Supplementary file 3 — Additional file 3: Fig. S3. rDKK3 alleviated neuroinflammation in the spinal cord induced by neuropathic pain. (a-c) Western blot results indicated that administration with rDKK3 reduced the pro-inflammatory cytokines level of IL-1β, IL-6 and TNF-α in the spinal cord caused by neuropathic pain (*p < 0.05, **p < 0.01, ***p < 0.001 compared with Sham + Vehicle group, #p < 0.05, ###p < 0.001, ###p < 0.0001 compared with SNI + Vehicle group, n = 6 in each group). (d-f) ELISA results showed that rDKK3 suppressed neuroinflammation in the spinal cord caused by neuropathic pain (***p < 0.001, ****p < 0.0001 compared with Sham + Vehicle group, ##p < 0.01, compared with SNI + Vehicle group, n = 6 in each group). [file 12974_2022_2495_MOESM3_ESM.pdf]

(a)

CD86

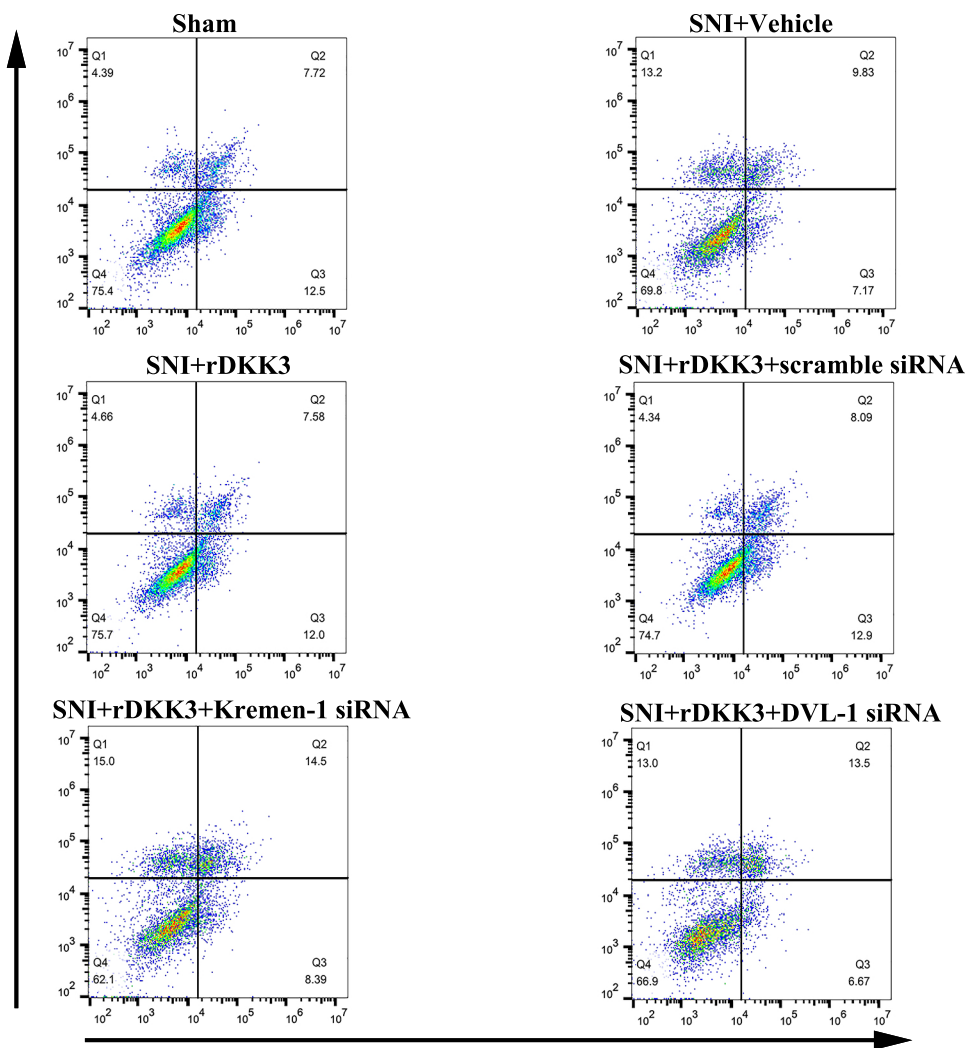

(b)

CD206

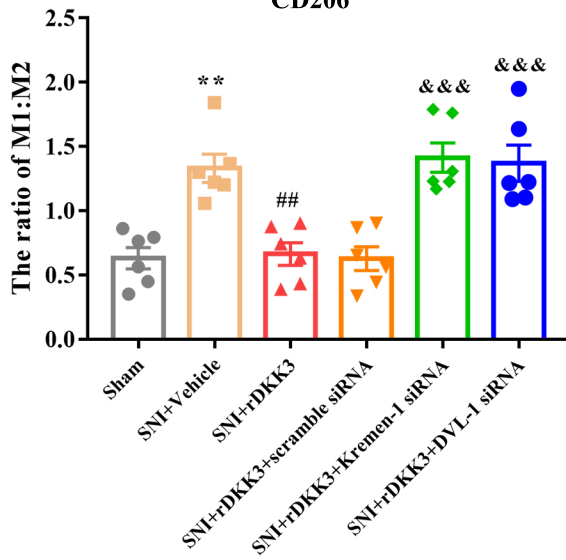

Supplement: Supplementary file 4 — Additional file 4: Fig. S4. The Flow cytometry result of the Sham group, SNI + Vehicle group, SNI + rDKK3 group, SNI + rDKK3 + scramble siRNA group, SNI + rDKK3 + Kremen-1 siRNA group, and SNI + rDKK3 + DVL-1 siRNA group. (a) Representative dot spot of flow cytometry for microglia. (b) Histogram showed that rDKK3 promoted the switch of microglia from M1 type to M2 type in the spinal cord of rats with neuropathic pain, while Kremen-1 siRNA and DVL-1 siRNA could abrogate the effect of rDKK3 on microglia polarization (**p < 0.01 compared with Sham group, ##p < 0.01 compared with SNI + Vehicle group, &&&p < 0.001 compared with SNI + rDKK3 group, n = 6 in each group). [file 12974_2022_2495_MOESM4_ESM.pdf]

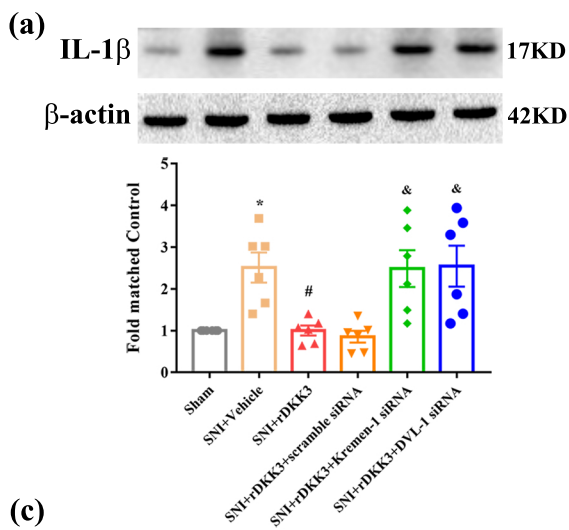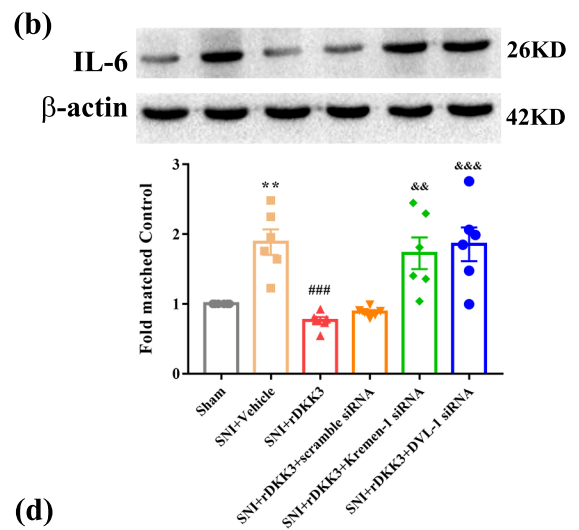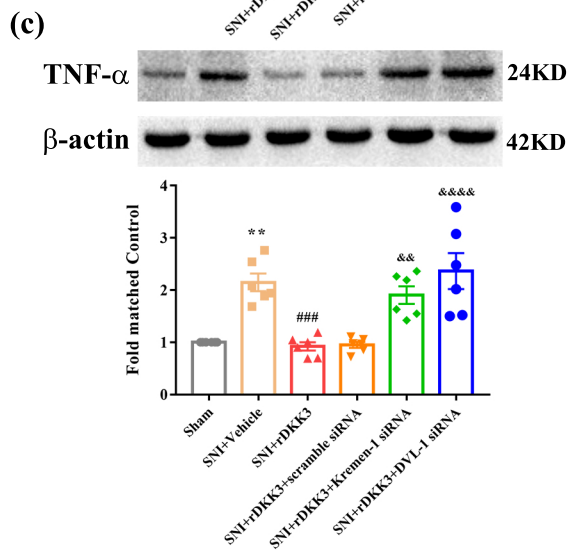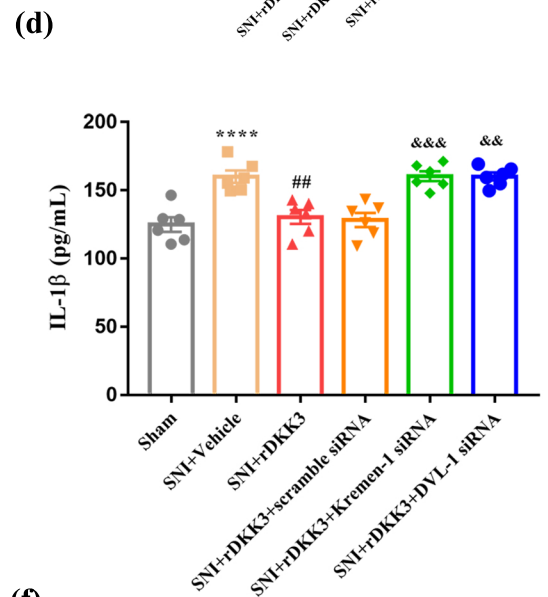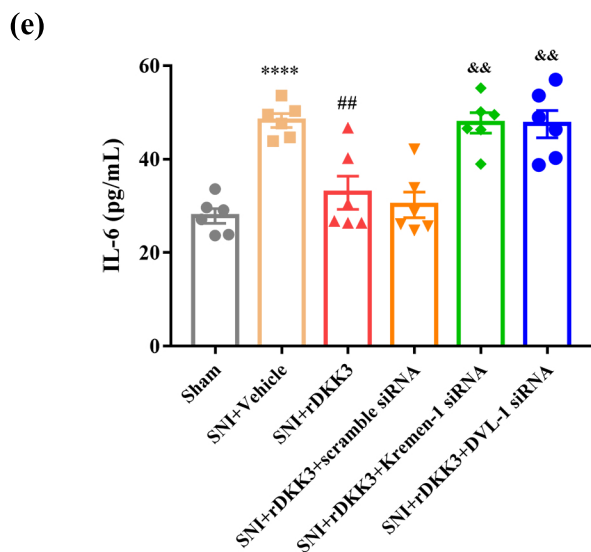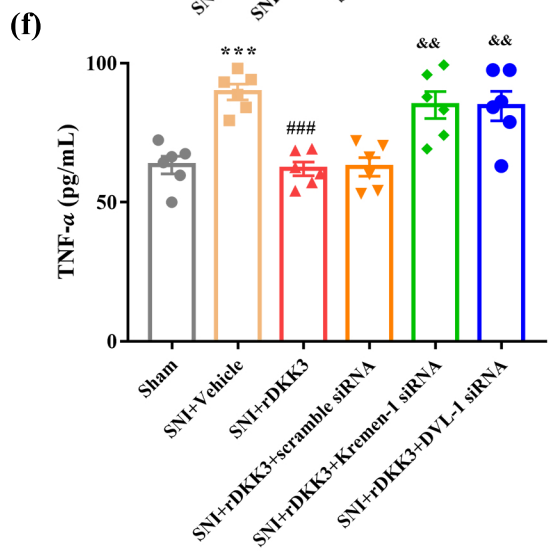

Supplement: Supplementary file 5 — Additional file 5: Fig. S5. The improved neuroinflammation caused by rDKK3 was abolished by Kremen-1 siRNA or DVL-1 siRNA in neuropathic pain rats. (a-c) Western blot results indicated that rDKK3 reduced the pro-inflammatory cytokines level of IL-1β, IL-6 and TNF-α in the spinal cord caused by neuropathic pain, while administration with Kremen-1 siRNA or DVL-1 siRNA up-regulated the decreased pro-inflammatory cytokines level of IL-1β, IL-6 and TNF-α in the spinal cord caused by neuropathic pain (*p < 0.05, **p < 0.01 compared with Sham group, #p < 0.05, ###p < 0.001 compared with SNI + Vehicle group, &p < 0.05, &&p < 0.01, &&&p < 0.001, &&&&p < 0.0001 compared with SNI + rDKK3 group, n = 6 in each group). (d-f) ELISA results showed that the improved neuroinflammation in the spinal cord caused by rDKK3 was abolished by Kremen-1 siRNA or DVL-1 siRNA in rats with neuropathic pain (***p < 0.001, ****p < 0.0001 compared with Sham group, ##p < 0.01, ###p < 0.001 compared with SNI + Vehicle group, &&p < 0.01, &&&p < 0.001 compared with SNI + rDKK3 group, n = 6 in each group). [file 12974_2022_2495_MOESM5_ESM.pdf]
